# A light-fostered supercapacitor performance of multi-layered ReS_2_ grown on conducting substrates†

**DOI:** 10.1039/d0na00901f

**Published:** 2021-02-15

**Authors:** Nitika Arya, Piyush Avasthi, Viswanath Balakrishnan

**Affiliations:** School of Engineering, Indian Institute of Technology, Mandi Himachal Pradesh 175005 India viswa@iitmandi.ac.in

## Abstract

The light-fostered supercapacitor performance introduces a new realm in the field of smart energy storage applications. Transition metal dichalcogenides (TMDCs) with direct band gap are intriguing candidates for developing a light-induced supercapacitor that can enhance energy storage when shined with light. Many TMDCs show a transition from a direct to indirect band gap as the layer number increases, while ReS_2_ possesses a direct band gap in both bulk and monolayer forms. The growth of such multi-layered 2D materials with high surface area on conducting substrates makes them suitable for smart energy storage applications with the ability to tune their performance with light irradiation. In this report, we present the growth of vertically aligned multi-layered ReS_2_ with large areal coverage on various conducting and non-conducting substrates, including stainless steel *via* chemical vapor deposition (CVD). To investigate the effect of light illumination on the charge storage performance, electrochemical measurements have been performed in dark and light conditions. Cyclic voltammetry (CV) curves showed an increase in the area enclosed by the curve, manifesting the increased charge storage capacity under light illumination as compared to dark. The volumetric capacitance value calculated from charging–discharging curves has increased from 17.9 F cm^−3^ to 29.8 F cm^−3^ with the irradiation of light for the as-grown ReS_2_ on a stainless steel plate. More than 1.5 times the capacitance enhancement is attributed to excess electron–hole pairs generated upon light illumination, contributing to the charge storage in the presence of light. The electrochemical impedance spectroscopy further augments these results. The high cyclic stability is attained with a capacitance retention value of 81% even after 10 000 repeated charging–discharging cycles.

## Abstract 2

The light-fostered supercapacitor performance introduces a new realm in the field of smart energy storage applications.

## Graphical abstract

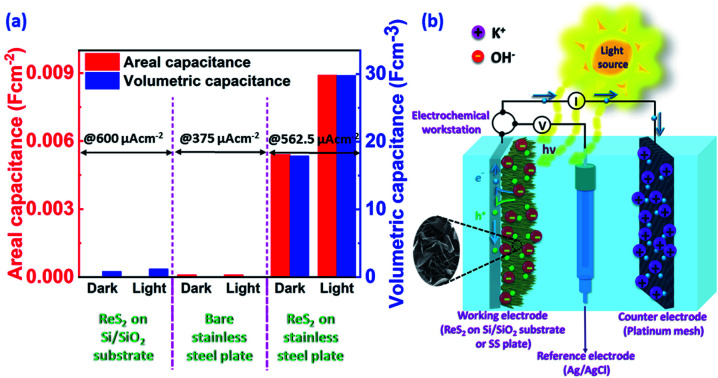

## 1. Introduction

Smart supercapacitors with the ability to charge when exposed to light is an attractive research area for developing next-generation energy storage devices.^1–12^ Light-sensitive direct band gap semiconductor materials with energy storage capacity are of great importance for such light-tunable supercapacitor performances. While these energy storage devices have gained enormous attention by researchers for various applications such as e-vehicles and portable electronic devices,^13–21^ the development of smart supercapacitors is in its infant stage. In particular, supercapacitors having high cyclic stability and high power density^22,23^ with the ability to tune their charge–discharge characteristics are of great significance for smart energy storage technologies. Technologies, such as solar cells, photo-catalysis and others based on solar energy, have gained immense attention due to their high conversion efficiency and low cost.^24–29^ Although a large number of applications has been explored using solar energy as a source, its potential in supercapacitor performance still remains untapped. Consequently, research articles that we have encountered are limited.^1–3,6,7,9,11,30^ Recently, efforts have been made to enable the development of photo supercapacitors by integrating the aforementioned aspects of smart energy storage, solar energy and light-sensitive semiconducting materials.^4,5,10,12,31–33^ Such hybridization between the energy conversion and energy storage materials resulted in the development of the photo supercapacitor by coupling dye-sensitized solar cells (DSSCs) with supercapacitors, where the dye molecules of DSSCs absorb and convert solar energy to electrical energy, and finally transfer it to the supercapacitor, which stores this energy.^10,31,34,35^ Such integrated systems possess lower overall efficiency in comparison with their individual counterparts. This is due to the fact that the overall efficiency of the pronounced integrated system relies upon the performance of the conversion process and storage process, thereby making the high-performance active materials an essential condition on both parts.^35^ The mismatch between energy generation and energy storage can result in lower efficiency. For instance, a storage device with higher capacity per charging cycle is available. However, if the conversion device is not that efficient, then it will lead to a mismatch or *vice versa*. This mismatch can arise due to the physical dimension mismatch.^35,36^ Also, the integration of such systems may increase the internal resistance, which further lowers the efficiency.^35,36^ However, this can be ameliorated using one electrode, which is proficient in both conversion and the storage process. Therefore, it would be advantageous to have the capability of producing photoelectrons and potential to store energy in a single material. Recently, such light-induced energy storage devices have been demonstrated in the UV and visible range for smart self-conditioned battery management applications using CNT-based electrode materials.^37^

Photo-charging ensues by the generation of electron–hole pairs and their storage within the material in the presence of light. Therefore, a low-cost material with moderate electrical conductivity will possess an optimum band gap. This allows a prolonged separation of charge carriers having high surface area to accumulate a large number of electrolyte ions at the interface, thus, increasing the capacitance is a prerequisite. By taking these aspects into consideration, we target the development of a low-cost, high surface area electrode material with an optimum band gap for photo supercapacitor energy applications by scrutinizing TMDCs. Most TMDCs are known to possess a direct band gap in their monolayer form that differs from their bulk counterparts and possess an indirect band gap.^38^ This acts as the main constraint for those applications where a high surface area is an indispensable factor. ReS_2_, owing to its direct band gap of 1.55 eV even in bulk form unlike other TMDCs, makes it a preeminent candidate for such applications.^39,40^ Due to the weak interlayer coupling commencing from the Peierls distortion of the 1T structure of ReS_2_, it behaves as decoupled monolayers both electronically and vibrationally, ensuing a very small change in the band structure when the layer number is increased.^40,41^ In fact, it has been reported that the photoluminescence (PL) intensity in ReS_2_ increases with an increase in the number of layers, making it a transcendent candidate for the aforementioned application.^40^ Although the supercapacitor performance of ReS_2_ has been studied recently, along with the solar driven charge storage properties,^42,43^ the light effect on the charge storage properties of direct band gap materials (such as ReS_2_ multi-layers grown on a conducting substrate) remains unexplored.

Here, we investigated the growth of vertically aligned multi-layered ReS_2_ directly on conducting and non-conducting substrates *via* CVD method, and explored their supercapacitor performance with light. The detailed microscopic and spectroscopic investigations confirmed the formation of pure ReS_2_ multi-layers having vertically grown flower-like morphology with large areal coverage. The charge storage performance has been examined using cyclic voltammetry (CV) and galvanostatic charge–discharge (GCD) on conducting, as well as non-conducting substrates as the current collector. The results confirm more than a 1.5-fold increase in the specific capacitance in the presence of light, as compared to the dark condition in the case of the conducting current collector. The contribution from both electrical double layer and pseudocapacitance has been observed in CV and GCD with 81% capacitance retention over 10 000 charge–discharge cycles.

## 2. Experimental section

### 2.1. CVD growth of ReS_2_ nanoflowers

ReS_2_ nanoflowers were synthesized in a Thermo Scientific tube furnace (Model Lindberg Blue M) *via* a lucid single step CVD method, using ammonium perrhenate powder (99.999% trace metals basis, Sigma Aldrich) and sulphur powder (325 mesh, 99.5%, Alfa Aesar) as the precursors at 600 °C growth temperature in atmospheric pressure. A quartz boat loaded with 30 mg of the ammonium perrhenate powder was positioned at the center of the furnace, and an alumina boat loaded with 200 mg of sulphur powder was placed at the outer edge of the heating zone such that the temperature of sulphur reaches between 180–200 °C when the growth temperature was attained at the center of the furnace. A clean substrate was loaded on top of the ammonium perrhenate-containing quartz boat. The tube with argon as the carrier gas was purged at a flow rate of 240 sccm for 15 minutes at room temperature before starting the growth. Initially, the temperature of the furnace at the center was raised to 600 °C in 20 minutes with a flow rate of 10 sccm argon gas, and held there for 45 minutes with a constant argon flow rate of 10 sccm. Eventually, the furnace was allowed to naturally cool down to room temperature once the growth was complete.

### 2.2. Characterizations

The microstructural characterization was carried out by using a field emission scanning electron microscope (FESEM)-FEI (now Thermo Fisher Scientific) – Nova NanoSEM 450 and high-resolution transmission electron microscope (HRTEM) – Tecnai G^2^ T20 S-TWIN, FEI (now Thermo Fisher Scientific) operated at 200 kV. The TEM sample was prepared by directly drop casting the extensively sonicated suspended solution of the ReS_2_ grown on Si/SiO_2_ substrate in acetone on a 3 mm carbon-coated copper grid. The crystal structure and phase purity of ReS_2_ were analyzed by using X-ray diffraction (Rigaku SmartLab diffractometer). A Horiba HR-Evolution Raman spectrometer was used to record the Raman and photoluminescence spectra. A Thermo Scientific Nexsa XPS System was used to detect the elemental composition, chemical state and electronic state of the elements present in the material. The optical absorption characteristics of the as-grown ReS_2_ on Si/SiO_2_ substrate was evaluated from diffuse reflectance spectroscopy (DRS) using a UV-Vis spectrophotometer (SHIMADZU-UV 2600) after calibrating it with a cleaned Si/SiO_2_ substrate as the reference.

### 2.3. Electrochemical measurements

Cyclic voltammetry (CV), galvanostatic charge–discharge (GCD) and electrochemical impedance spectroscopy (EIS) were performed using a Biologic VSP electrochemical workstation in 1 M aqueous KOH. All of the measurements were carried out in a standard three electrode configuration, where the as-grown ReS_2_ on a stainless steel plate was directly used as the working electrode, whereas a small amount of silver paste was applied in the case of ReS_2_ on a Si/SiO_2_ substrate for making contact, platinum mesh as the counter electrode and Ag/AgCl as the reference electrode, respectively. A 150 W xenon lamp (Holmarc) ranging from 200 to 2500 nm wavelength was used as the light source for studying the light radiation effect on developed materials towards supercapacitor application.

## 3. Results and discussion

**Fig. 1:**
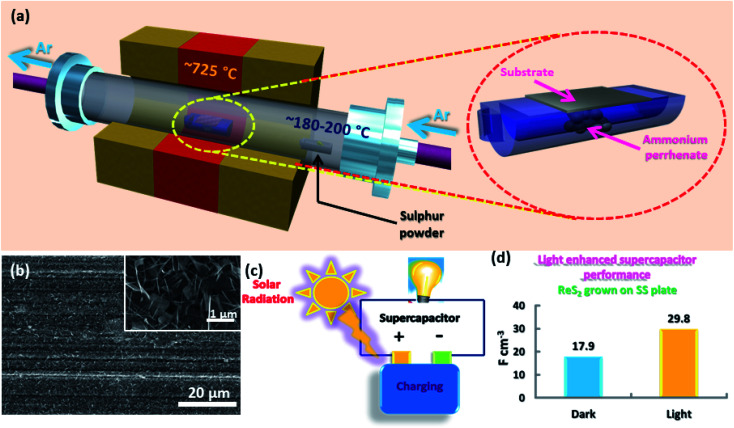
Essence of the story: (a) schematic representation of the ReS_2_ growth *via* CVD, (b) representative SEM micrograph of ReS_2_ grown on a SS plate with very high coverage; a high magnification image showing the vertically aligned microstructure of ReS_2_ is presented in the inset, (c) illustration of the supercapacitor charging on the illumination of light, and (d) bar chart shows around 1.5-fold enhancement in the volumetric capacitance of CVD-grown ReS_2_ on a SS plate under light illumination.

Vertically aligned ReS_2_ was grown on a 3 × 1 cm^2^ conducting stainless steel plate (SS plate) by using ammonium perrhenate and sulphur powder as the Re and S precursors, respectively. The CVD growth scheme is illustrated in Fig. 1(a). The used precursors thermally decompose and react on the SS plate surface in the vapor form to produce ReS_2_ with the aid of the Ar carrier gas supply. The areal coverage of the as-grown ReS_2_ on a SS plate was examined using FESEM. The FESEM micrograph, as shown in Fig. 1(b), manifests the high coverage growth of ReS_2_ on the SS plate. The corresponding high magnification FESEM micrograph shows the vertically oriented microstructure of ReS_2_ with flower petal-like morphology having around 10 nm thickness and lateral size varying from 500 nm to 1.6 μm, as shown in the inset of Fig. 1(b). ReS_2_ on the SS plate with a high surface area was then used as a light-assisted charge-storage electrode material for the supercapacitor application under light illumination. A general illustration is presented in Fig. 1(c). ReS_2_ grown on a SS plate showed a considerable (∼1.7-fold) increase in the specific capacitance under light illumination. This is depicted in the form of a bar chart in Fig. 1(d).

**Fig. 2:**
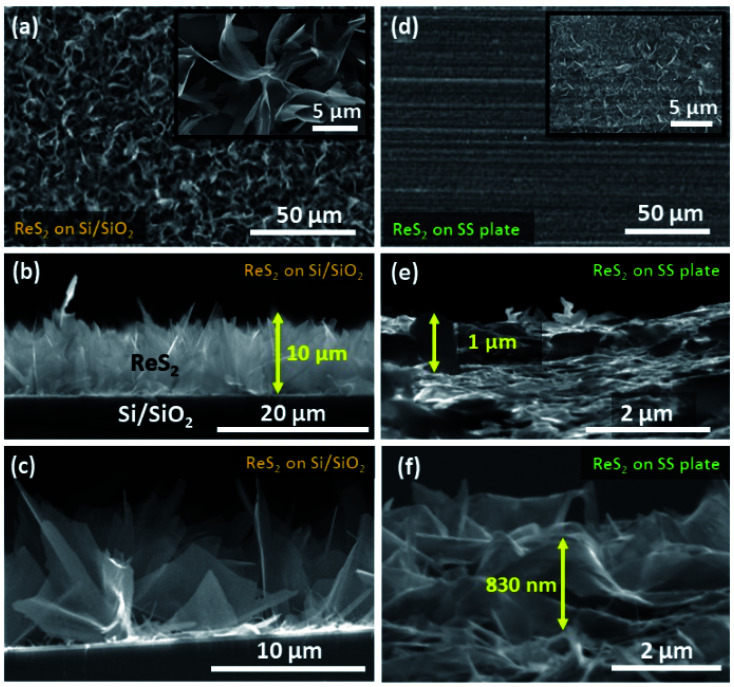
ReS_2_ morphology and height identification on the Si/SiO_2_ and SS plate: (a) low magnification planar FESEM image of ReS_2_ grown on the Si/SiO_2_ substrate; corresponding high magnification image having the vertically aligned high surface area flower-like morphology is shown in the inset, (b and c) cross-sectional FESEM images showing the height of the vertically aligned ReS_2_ and individual flakes, (d) low magnification planar FESEM image of ReS_2_ grown on a SS plate; the representative high magnification image is shown in the inset, (e and f) cross-sectional FESEM images showing the height of the vertically aligned ReS_2_ and individual flakes.

The controlled growth of ReS_2_ was performed on four different substrates, including both conducting and non-conducting substrates. The low magnification FESEM micrographs for ReS_2_ grown on Si/SiO_2_ and SS plate are presented in Fig. 2(a) and (d), and that of ReS_2_ grown on FTO coated glass and ITO coated glass are presented in Fig. S1 (ESI†), showing the variation in the areal coverage of the ReS_2_ growth. This indicates the substrate dependence growth of ReS_2_. This kind of substrate-dependent ReS_2_ growth has already been explored by various researchers in their reports, where the energy barriers provided by the substrate to the adatoms and binding strength of the adatoms onto the substrate play a key role.^44,45^ The corresponding high magnification FESEM images are shown in the inset of Fig. 2(a) and (d), showing the vertically oriented petal-like morphology, which are well connected with other ReS_2_ flakes. There are several mechanisms proposed for the vertically oriented growth of the multi-layer TMDCs. For example, the distorted 1T structure of ReS_2_ (along with its weak interlayer coupling) is believed to promote the vertically oriented asymmetric growth.^46^ The coalescence-based mechanism was also proposed for the vertical orientation evolution in the dense growth of multi-layered 2D materials.^47^ Nevertheless, the detailed growth mechanism based on high supersaturation during the CVD growth seems to unambiguously explain the present observations. It has been reported that the vertical growth is induced when the surface interaction between the nucleating layer and substrate is favorable at high supersaturation conditions.^48^ It should be noted that the mechanisms based on the distorted 1T structure of ReS_2_ and coalescence may not explain the observed substrate dependence over the vertically oriented growth. To determine the thickness or height of the vertically grown ReS_2_, cross-sectional FESEM imaging was carried out, as shown in Fig. 2(b, c) and (e, f). This height was used for calculating the volumetric capacitance of ReS_2_ grown on the Si/SiO_2_ substrate and SS plate. The cross sectional SEM image of ReS_2_ grown on Si/SiO_2_ clearly reveals the vertical growth of ReS_2_ flakes with an average height of 10 μm. Similarly, the cross sectional SEM images recorded from ReS_2_ on SS has been used to estimate the average thickness of ReS_2_. However, it was difficult to determine the exact thickness of ReS_2_ from the cross-sectional imaging due to the uneven SS surface and indistinctness of the depth of ReS_2_ grown on the SS plate. Unlike the ReS_2_ growth on Si/SiO_2_, the growth on a SS plate was observed for all sides of the SS plate, including the edges. Nevertheless, the high magnification SEM images recorded from different regions indicate that the height of the individual ReS_2_ flakes is in the range of 0.5 μm to 1 μm. Representative SEM images of the individual ReS_2_ flakes are shown in Fig. 2(f). Since the height of the nanostructure is inversely proportional to the volumetric capacitance, we considered the height of ReS_2_ grown on the SS plate as 3 μm, instead of 1 μm or less, to be on the safer side and to provide an underestimated value for the volumetric capacitance.

**Fig. 3:**
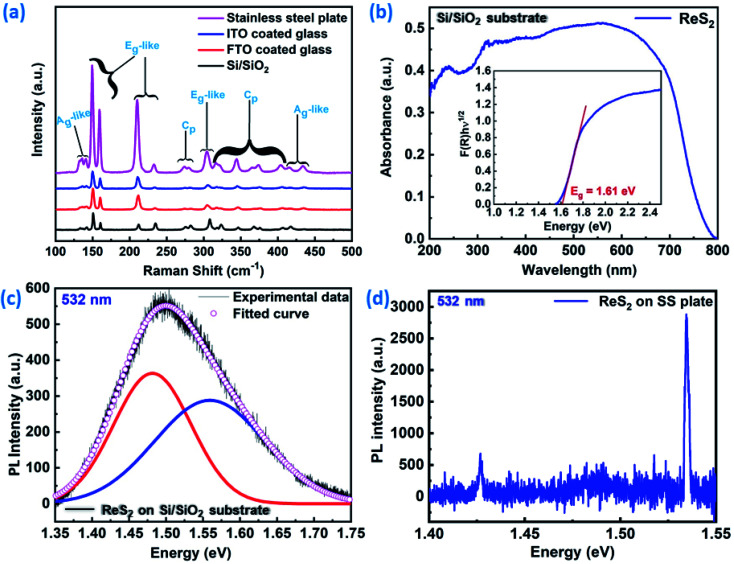
Phase purity detection and band gap measurement: (a) Raman spectra corresponding to ReS_2_ grown on Si/SiO_2_, FTO-coated glass, ITO-coated glass and SS plate, showing the phase pure nature of the as-grown ReS_2_, (b) UV-visible absorption spectra and Tauc plot, showing the band gap of ReS_2_ grown on the Si/SiO_2_ substrate. Photoluminescence spectra taken at an excitation wavelength of 532 nm for the band gap measurement for ReS_2_ grown on a (c) Si/SiO_2_ substrate and (d) SS plate.

Raman spectroscopy was used to confirm the growth of ReS_2_ on all of the substrates using an excitation wavelength of 532 nm laser. Contingent upon the in-plane and out-of-plane vibrations of the atoms of Re and S, various Raman modes have been identified. The peaks located between 135–142 cm^−1^ corresponds to the A_g_-like modes for the out-of-plane vibrations of the Re atoms, 149–234 cm^−1^ represents the E_g_-like modes of the Re atoms in-plane vibrations, 274–283 cm^−1^ corresponds to the C_p_ modes for the in-plane and out-of-plane vibrations of the atoms of Re and S, 304–312 cm^−1^ corresponds to the E_g_-like modes of the in-plane vibrations of the S atoms, 316–407 cm^−1^ represents the C_p_ modes for the in-plane and out-of-plane vibrations of the S atoms, and 415–435 cm^−1^ corresponds to the A_g_-like modes for the out-of-plane vibrations of the S atoms, respectively. A detailed description is presented in Table ST1 (ESI†). The Raman modes present in the spectra (Fig. 3) are congruent with earlier reported literature values for a multi-layered ReS_2_.^49,50^ Our results are consistent and very close to the reported values of He *et al.*, who used a 532 nm laser as the excitation wavelength.^49^ It is evident from Fig. 3(a) that we have successfully grown ReS_2_ on a variety of different substrates. For gaining more insight with respect to the electronic structure, UV-visible and photoluminescence spectra were obtained and analyzed. The UV-visible spectroscopy confirms the band gap of 1.61 eV, as shown in Fig. 3(b). Fig. 3(c) represents the PL spectra obtained by employing the excitation wavelength laser of 532 nm for ReS_2_ grown on a Si/SiO_2_ substrate. Multiple peak fitting was carried out using Gaussian profile curves for these PL spectra. These fitted curves altogether give rise to two peaks at 1.47 eV and 1.57 eV. Similar peaks were observed for ReS_2_ grown on a SS plate with a small shift of 0.04 eV (Fig. 3(d)) with considerable enhancement intensity. These two peaks at different energies correspond to different optical transitions, direct excitonic states. These values are consistent with the values claimed by Ho *et al.* in their report.^51^ Also, Tongay *et al.* reported an increase in the PL intensity from the monolayer to the bulk ReS_2_ sample, which can be very beneficial for the high surface area light-assisted applications.^40^ The investigated optical absorption and emission behaviors suggest that the CVD grown semiconductor ReS_2_ with multiple transition states are suitable for light-tunable energy storage applications.

**Fig. 4:**
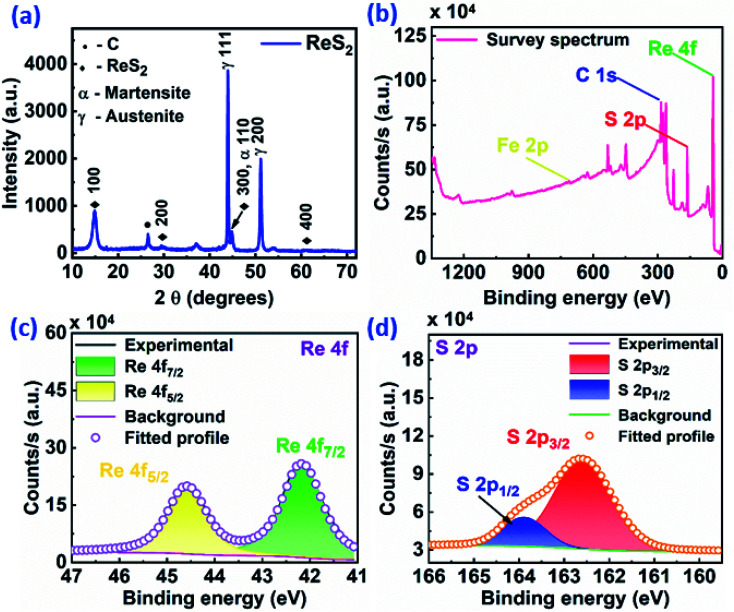
Crystallographic identification and elemental composition analysis of ReS_2_ on the SS plate: (a) X-ray diffraction pattern of ReS_2_ flakes, indicating their crystalline nature with the preferred orientation of the (*h*00) family reflection. XPS spectra of: (b) survey, (c) Re, and (d) S, showing the elemental composition and chemical states of the as-grown ReS_2_ on the SS plate.

In order to gain more insight into the ReS_2_ microstructure, TEM imaging was carried out for ReS_2_ grown on a Si/SiO_2_ substrate. The bright-field TEM micrograph in Fig. S2(a)† unveils the electron transparent ultrathin structure of ReS_2_ nanoflowers at low magnification. Fig. S2(b)† shows the HRTEM image, manifesting that the as-grown ReS_2_ is crystalline in nature. The measured lattice fringes with a *d*-spacing of 0.53 nm is attributed to the (010) plane of ReS_2_. The corresponding selected area electron diffraction (SAED) with spot pattern shown in the inset further endorses its single crystalline nature. The HRTEM micrograph presented in Fig. S2(c)† provides an impression regarding the number of layers of the as-grown nanoflowers of ReS_2_ with an interlayer spacing of 0.6 nm. The average number of layers ranges between 6 to 12. Furthermore, to verify the single crystalline nature of ReS_2_, X-ray diffraction analysis was carried out, as shown in Fig. S2(d),† which indicates the presence of five prominent peaks. The peak positioned at 68.97° corresponds to Si from the Si/SiO_2_ substrate, whereas the peaks at 14.41°, 29.15°, 44.45° and 60.67° are attributed to the (100), (200), (300) and (400) crystallographic planes of a triclinic (Anorthic) ReS_2_ crystal structure (JCPDS File no. 82-1379), respectively. X-ray diffraction analysis was carried out for the ReS_2_-grown SS plate as well to find out the crystalline nature and purity of the as-grown sample. This is shown in Fig. 4(a), which indicates the similar single crystalline nature of the ReS_2_ grown on the SS plate, along with some additional peaks for the SS plate. Only the (*h*00) family reflections are observed in both cases with the absence of all other (*hkl*) reflections, thereby implying the highly oriented single crystalline nature of these ReS_2_ nanostructures.

Fig. 4(b) and (c) shows the XPS results, which appraise the elemental composition and their chemical states in the ReS_2_ nanoflowers grown on a SS plate. Fig. 4(b) displays the survey spectrum of the as-grown ReS_2_, which shows the presence of three elements, Re and S from ReS_2_ and Fe from the SS plate. The charge correction referencing is done by setting the C 1s line to 284.8 eV, which appears due to adventitious carbon contamination. The XPS spectrum for Re possesses two predominant peaks positioned at binding energies of 42.18 eV and 44.58 eV, attributed to the Re 4f_7/2_ and Re 4f_5/2_ core levels, respectively, as shown in Fig. 4(c). These are the characteristic peaks of the Re^4+^ oxidation state. The XPS spectrum of S is shown in Fig. 4(d) and possesses two peaks positioned at binding energies of 162.58 eV and 163.88 eV, which are ascribed to the 2p_3/2_ and 2p_1/2_ core levels, respectively. These are the characteristic peaks for the sulphide (S^2−^) ions. All of the peaks are consistent with previously reported literature values.^46^ Furthermore, the elemental composition and their chemical states were confirmed in ReS_2_ grown on the Si/SiO_2_ substrate as well. The corresponding XPS results are shown in Fig. S3 in ESI† with similar peak features with a small shift of 0.5–0.6 eV.

**Fig. 5:**
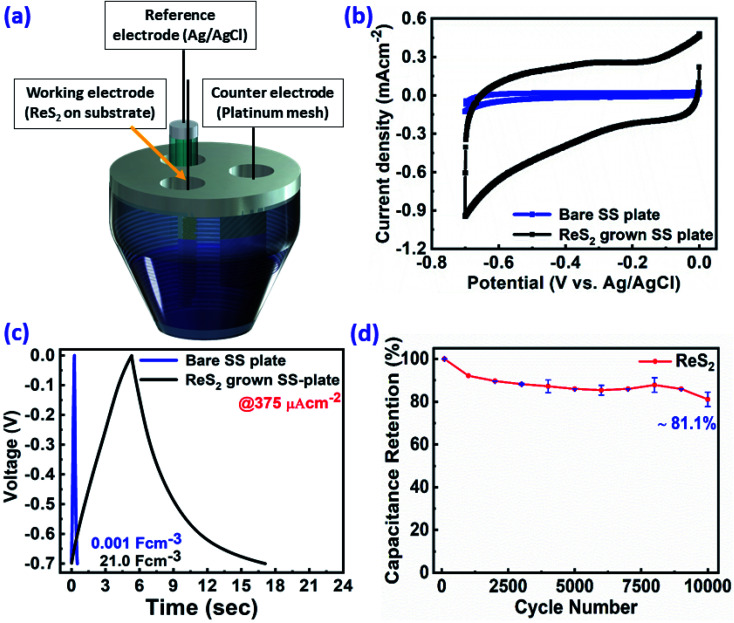
Electrochemical supercapacitor performance of the as-grown ReS_2_ on a SS plate (black), as compared to the bare SS plate (blue): (a) three electrode system used for the electrochemical measurements, (b) cyclic voltammogram taken at 100 mV s^−1^ scan rate, (c) galvanostatic charge–discharge cycles at 375 μA cm^−2^ showing the superior charge-storage capacity of ReS_2_ grown on a SS plate in terms of the area enclosed by the CV curve and specific capacitance value calculated from the discharging time in terms of the volumetric capacitance, and (d) capacitive retention plotted against the number of cycles showing ∼81% capacitance retention over 10 000 charge–discharge cycles.

In order to evaluate the charge storage performance of the as-grown ReS_2_ electrode, electrochemical measurements of the bare SS plate and ReS_2_ grown on the SS plate have been carried out in a three-electrode configuration, as shown in Fig. 5(a) using CV, GCD and EIS in 1 M KOH aqueous electrolyte. Cyclic voltammetry was carried out in the potential range of 0 to −0.7 V at various scan rates, ranging from 20 mV s^−1^ to 500 mV s^−1^. The comparative CV curves of the bare SS plate and ReS_2_ grown on the SS plate taken at 100 mV s^−1^ are shown in Fig. 5(b), manifesting that ReS_2_ grown on the SS plate shows more charge-storage capacity as compared to the bare SS plate, as reflected from the area enclosed by the CV curves. The following equation was employed for calculating the areal capacitance from the CV curves:^52^1
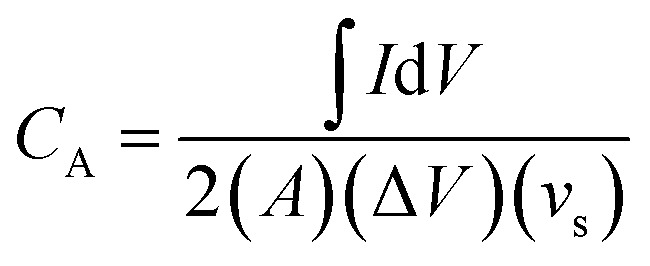
where 
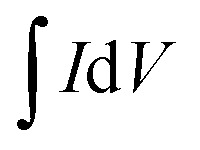
 is the area enclosed by the CV curve, *A* is the dipped area of the working electrode, Δ*V* is the potential window and *v*_s_ is the scan rate in mV s^−1^. For calculating the volumetric capacitance, the following equation was used:2
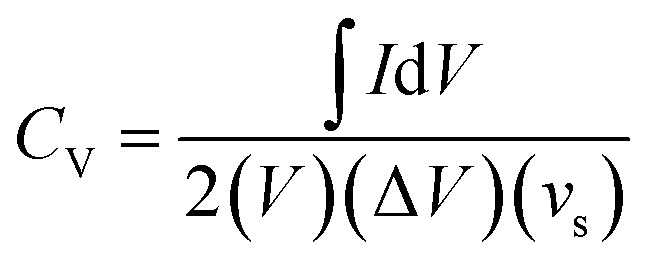
where *V* is the volume of material present in the dipped area of the working electrode estimated by considering the actual thickness and geometrical area using the following equation:

3 *V* = *A* × *t*

The thickness of ReS_2_ grown on the SS plate was taken as 3 μm based upon the cross-sectional FESEM imaging done for measuring the height of ReS_2_ grown on the SS plate. A volumetric capacitance value of 0.448 mF cm^−3^ was calculated for the bare SS plate and 3033 mF cm^−3^ for ReS_2_ grown on the SS plate. The shape of the CV curves highlights the primary contribution of EDLC, along with a slight contribution from the pseudocapacitance. The comparative GCD curves acquired at 375 μA cm^−2^ current density in Fig. 5(c) clearly reflect the increased charge storage capability of ReS_2_ grown on the SS plate with a volumetric capacitance value of 21 066 mF cm^−3^ as compared to the bare SS plate with 1.12 mF cm^−3^. The following equation was used for calculating the areal capacitance from the GCD curves:^53,54^4
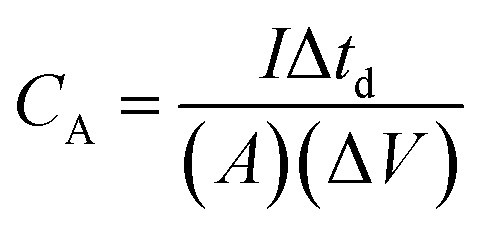
where *I* is the applied current, Δ*t*_d_ defines the discharging time, *A* is the dipped area of the working electrode, and Δ*V* is the potential window. For calculating the volumetric capacitance, the following equation was used:5
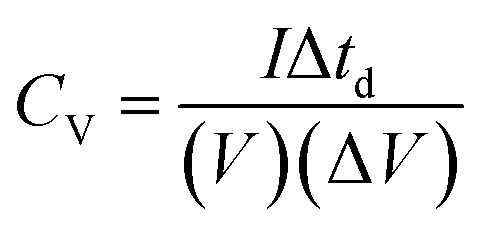


The ReS_2_ on the SS plate electrode was tested for cyclic stability, and it was found that the electrode material exhibits excellent stability with the capacitance retention of 81% over 10 000 GCD cycles, as shown in Fig. 5(d). The observed high capacitive retention is mainly due to direct CVD growth, which provides better mechanical integrity and adhesion of the electrode material over the substrate. The detailed electrochemical measurements for ReS_2_ grown on the SS plate are shown in Fig. S4 (ESI†). In addition, to gain insight into the electrochemical active surface area of ReS_2_ on the SS plate, we performed cyclic voltammetry experiments in a potential window of −0.1 V to −0.2 V (non-faradaic region) at different scan rates, starting from 20 mV s^−1^ to 500 mV s^−1^. This is shown in Fig. S5 (ESI†).^55–57^ The ECSA thus calculated was found to be 56.4 cm^2^. This manifests that a high surface area of 56.4 cm^2^ is provided for charge storage by the beautiful vertically aligned microstructure of ReS_2_ just within a small geometrical area of 0.8 cm^2^. Also, the electroactive mass of the as-grown ReS_2_ on the SS plate was determined by performing a back calculation. This was done by taking the density and volume (thickness × area) of ReS_2_ grown on a SS plate into consideration using the simple formula of *M* = *D* × *V*. The electroactive mass of the electrode thus calculated came out to be around 1.8 × 10^−3^ g or 1.8 mg.

**Fig. 6:**
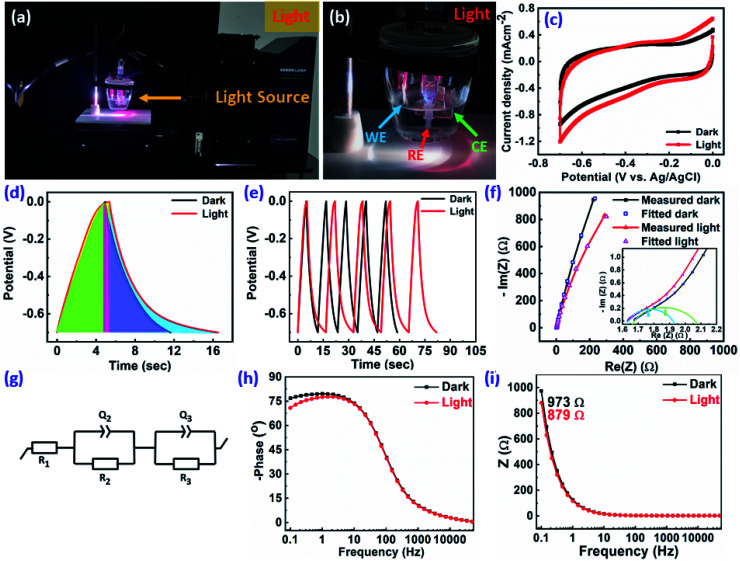
Light-induced improved electrochemical supercapacitor performance of the as-grown ReS_2_ on a SS plate: (a and b) photographs of the electrochemical measurement setup showing the path of the light source being irradiated on the working electrode and the ReS_2_ grown on the working electrode facing towards the light source, (c) cyclic voltammogram at 100 mV s^−1^ scan rate, (d) galvanostatic charge–discharge cycles at 562.5 μA cm^−2^ current density, (e) reversible nature of the light effect on the charging–discharging cycle is shown by plotting five GCD cycles (@562.5 μA cm^−2^ current density) in dark and light, (f) Nyquist plot measured and fitted, (g) equivalent circuit model used for fitting, (h) Bode phase angle plot and (i) Bode phase modulus plot for light (200–2500 nm (red)) and dark (black) conditions, showing the enhancement in the charge-storage capacity of ReS_2_ grown on a SS plate.

The ReS_2_ grown on a SS plate was further examined for light-induced supercapacitor performance in 1 M KOH aqueous electrolyte in the same potential window of 0 V to −0.7 V. The corresponding photographs of the electrochemical measurement setup used for examining the light-induced supercapacitor performance of our electrode material are shown in Fig. 6(a) and (b). During the light-induced electrochemical measurements, the side of the working electrode possessing ReS_2_ is kept facing towards the light source. The CV curves show an increase in the area enclosed by the curve on light illumination, as shown in Fig. 6(c), indicating the increased charge-storage capacitance on light illumination. Specific capacitance values of 4600 mF cm^−3^ and 3033 mF cm^−3^ were calculated with and without light illumination, respectively, in terms of the volumetric capacitance, and a 1.5-fold enhancement in the specific capacitance under light illumination has been observed. Fig. 6(d) shows the GCD curves for the sample of ReS_2_ grown on a SS plate in dark and light conditions obtained at a current density of 562.5 μA cm^−2^, indicating the increased discharging time upon light illumination. The detailed electrochemical measurements for ReS_2_ grown on a SS plate in light are introduced in Fig. S6 (ESI†). The delayed discharging observed under light exposure indicates the role of light. The specific capacitance values calculated from the GCD curves were found to be 17 946 mF cm^−3^ in the dark and 29 839 mF cm^−3^ upon illumination with light. This clearly reveals the effect of light on the supercapacitor charge storage. The light effect on the charging–discharging cycle was found to be reversible, and five GCD cycles in the dark (black) and light (red) are shown in Fig. 6(e). However, this effect of light on the charge storage capacity of the electrode material is much more prominent at lower current density, as shown in Fig. S7 (ESI†), where a ∼3-fold enhancement in the specific capacitance value is observed. However, the electrode polarization at lower current density is more pronounced beyond −0.5 V. It should be noted that the charging is primarily driven electrically, while the light provides additional charge carriers and more importantly, delays the discharging process. We believe that this is the outcome of the retarded discharging process in the presence of light irradiation, where the electrode discharge process is competing with the generation of more photogenerated electrons due to the light absorption process. The same is evident from the EIS studies, as shown in Fig. 6(f). This further validates the enhanced supercapacitor performance in terms of the decreased resistance values. The solution resistance shows a negligible change on light illumination in the high frequency region, as signified in the inset of Fig. 6(f). However, it can clearly be seen from Fig. 6(f) that the radius of the arc in the Nyquist plot under light illumination is noticeably reduced, which is the signature of the charge transfer resistance (CTR), indicating that the enhanced electron–hole pair separation and charge carrier flow are responsible for lowering the CTR. To better comprehend it, an equivalent circuit is proposed to fit the experimental data (Fig. 6(g)), as follows:6
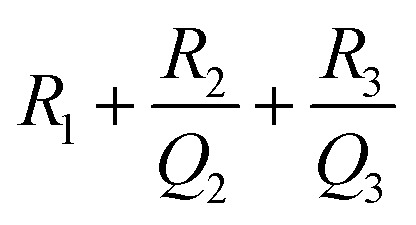
where *R*_1_ corresponds to the electrolyte (solution) resistance and can be evaluated as the intercept on the real axis in the high frequency region, *R*_2_ denotes the charge transfer resistance at the electrode–electrolyte interface, while *Q*_2_ (constant phase element) denotes the electrical double layer capacitance and is signified by the distorted semicircle in the mid-frequency region. In addition, *R*_3_ represents the recombination charge transfer resistance at the electrode–electrolyte interface and *Q*_3_ (constant phase element) denotes the chemical/faradaic capacitance at the electrode–electrolyte interface, and they are mainly associated with the low frequency region.^65^ The model fits the experimental data considerably, as can be seen in Fig. 6(f), and Table 1 summarizes the simulated equivalent circuit parameters thus obtained. *R*_1_ in the high frequency region shows a negligible difference after light illumination, *R*_2_ in the mid-frequency region shows a small difference, whereas *R*_3_ in the low frequency region shows a considerable difference. This shows that the ample reduction in the resistance is mainly observed in the low frequency region and at longer time duration, stabilizing the electron–hole pairs for more time. This leads to sluggish discharging kinetics. In addition to this, a decrease in *Q*_2_ signifies that the double layer capacitance is decreasing at the electrode–electrolyte interface, whereas the increase in *Q*_3_ indicates enhanced pseudo capacitance. The impedance of the constant phase element is given by:7
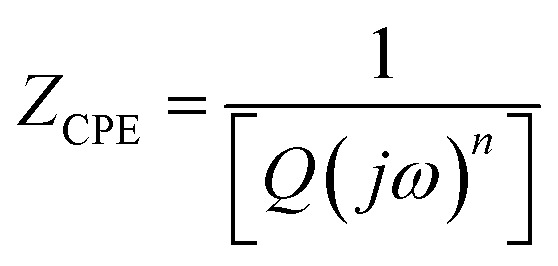
where *Q* represents a frequency-independent constant, *ω* represents the radial frequency, and the exponent *n* signifies the correction factor and can vary from −1 to 1. It performs as a pure inductor when *n* = −1, pure resistor when *n* = 0, and pure capacitor when *n* = 1. In the present case, *n*_2_ and *n*_3_ are approaching 1, but are not equal to one, thereby confirming the pseudocapacitive behavior.

**Table 1:** Equivalent circuit parameters for electrochemical impedance spectroscopy in the dark and upon light illumination

Condition	Parameters						
	*R*_1_ (Ω)	*Q*_2_ (F s^*n*−1^)	*n*_2_	*R*_2_ (Ω)	*Q*_3_ (F s^*n*−1^)	*n*_3_	*R*_3_ (kΩ)
Dark	1.66	9.25 × 10^−3^	0.65	0.41	1.52 × 10^−3^	0.90	12.000
Light	1.63	4.55 × 10^−3^	0.73	0.31	1.65 × 10^−3^	0.89	4.779

**Fig. 7:**
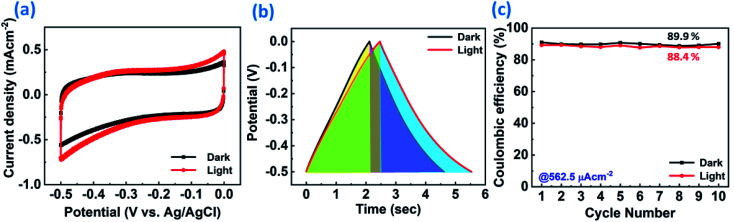
Electrochemical supercapacitor performance of the as-grown ReS_2_ on a SS plate in dark and light conditions in a reduced potential range (0 to −0.5 V) to avoid the electrode polarization effect: (a) comparative cyclic voltammogram at a scan rate of 100 mV s^−1^ in dark and light, (b) comparative galvanostatic charge–discharge cycles at a current density of 562.5 μA cm^−2^ in dark and light, (c) comparative coulombic efficiency plotted as a function of cycle number in dark and light.

Fig. 6(h) represents the Bode phase angle plot. The Bode phase angle is reduced in the presence of light, which is indicative of the slower charge transfer process. This reflects the slowing down of kinetics, in addition to the deviation from an ideal supercapacitor (EDL) behavior towards a pseudocapacitor behavior. The same is evident from the delayed discharging time observed in the GCD curves. The Bode modulus further strengthens this concept, where the impedance is decreased from 973 Ω to 879 Ω in the low frequency region upon light illumination, as shown in Fig. 6(i). The Bode modulus in the low frequency region indicates the solution resistance, interfacial resistance and charge transfer resistance cumulatively.^58^ This reduction in the resistance could be explained by the decreased charge transfer resistance after light illumination, as evident from the Nyquist plot (Fig. 6(f)), due to the generation of additional charge carriers contributing to enhanced charge storage. The sluggish kinetics is also evident from the modulus plot, and the deviation is observed only in the low frequency region, but not in the mid- and high frequency regions. The coulombic efficiency was calculated for ReS_2_ grown on a SS plate in both dark and light conditions, as shown in Fig. S8 (ESI†), proclaiming around 77.8% coulombic efficiency in the dark and around 71.3% in light. Since a considerable electrode polarization was observed at a potential higher than −0.5 V, as evident from Fig. 6(d), we limit the potential range for CV and GCD from 0 to −0.5 V, as shown in Fig. 7(a) and (b), respectively. The coulombic efficiency was found to be ∼90% in the dark and ∼88% in the light, as presented in Fig. 7(c). The detailed electrochemical measurements are shown in Fig. S9 (ESI†). It is a general practice to restrict the potential range of the charge discharge curves to avoid such undesirable and detrimental effect of the electrode polarization.^59–61^

**Fig. 8:**
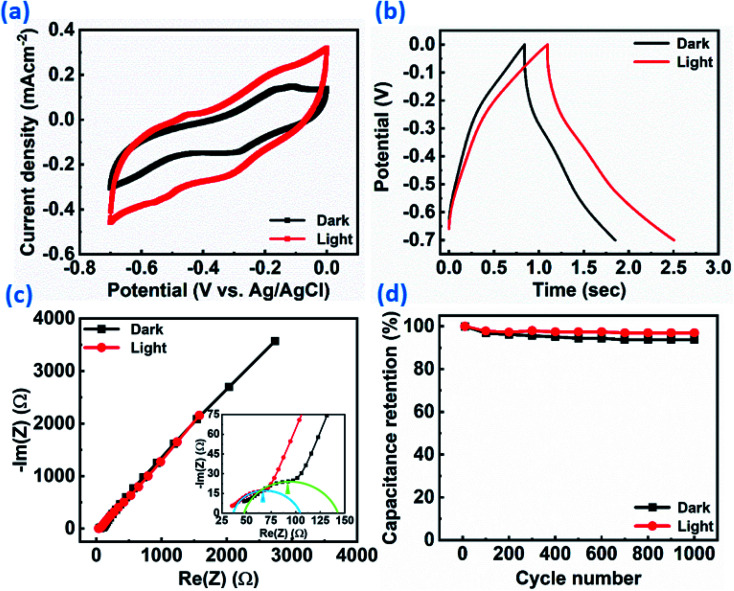
Light-induced improved electrochemical supercapacitor performance of the as-grown ReS_2_ on the Si/SiO_2_ substrate: (a) cyclic voltammogram at 100 mV s^−1^ scan rate, (b) Galvanostatic charge–discharge cycles at 600 μA cm^−2^ current density, (c) Nyquist plot, (d) capacitance retention after 1000 GCD cycles showing the cyclic stability in light (200–2500 nm (red)) and dark (black), showing the enhancement in the charge-storage capacity of ReS_2_ grown on the Si/SiO_2_ substrate.

**Fig. 9:**
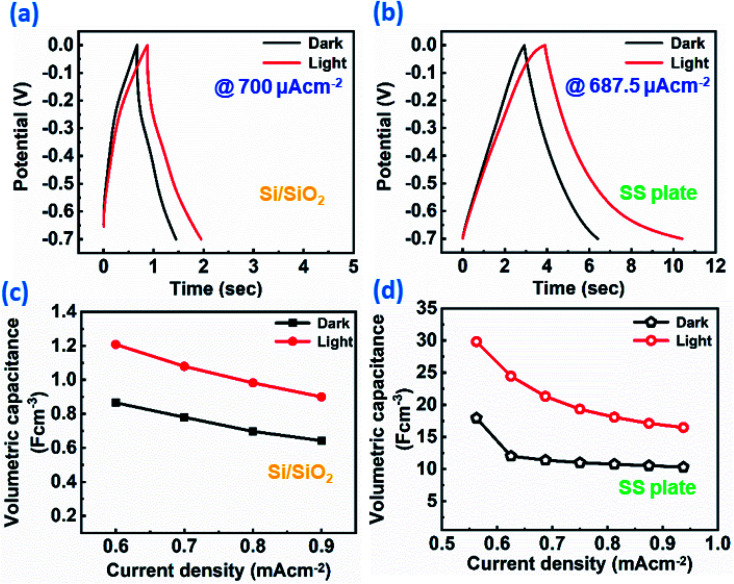
Light-induced improved electrochemical supercapacitor performance of the as-grown ReS_2_ on Si/SiO_2_ and SS plate in terms of the rate capability: (a) galvanostatic charge–discharge cycles at 700 μA cm^−2^ current density for ReS_2_ on the Si/SiO_2_ substrate, (b) galvanostatic charge–discharge cycles at 687.5 μA cm^−2^ current density for ReS_2_ on the SS plate, (c) rate capability of ReS_2_ grown on the Si/SiO_2_ substrate in terms of the volumetric capacitance as a function of the current densities, (d) rate capability of ReS_2_ grown on the SS plate in terms of the volumetric capacitance as a function of the current densities in the dark (black) and light (200–2500 nm (red)), showing the enhancement in the charge-storage capacity of ReS_2_ grown on the Si/SiO_2_ substrate and SS plate at all current densities.

Furthermore, the ReS_2_ grown on the Si/SiO_2_ substrate was examined for the light-induced supercapacitor performance in 1 M KOH aqueous electrolyte in the same potential window of 0 V to −0.7 V. The similar trend of the increase in the area enclosed by the CV curve on light illumination was exhibited by the ReS_2_ on the Si/SiO_2_ substrate. This is shown in Fig. 8(a), indicating the increased charge-storage capacitance on light illumination. The volumetric capacitance values of 592 and 749 mF cm^−3^ were calculated without and with light illumination, respectively, and approximately 1.2 times of enhancement in the specific capacitance under light illumination has been observed. The volumetric capacitance was calculated by considering the height of the vertically grown ReS_2_ as 10 μm as determined by the cross-sectional FESEM imaging multiplied by the geometrical area of the working electrode dipped in the electrolyte by using eqn (3). The GCD curves for ReS_2_ grown on Si/SiO_2_ substrate in dark and light conditions obtained at a current density of 600 μA cm^−2^ indicate the increased discharging time upon light illumination, as shown in Fig. 8(b). The specific capacitance values calculated from the GCD curves were found to be 865 mF cm^−3^ in dark and 1208 mF cm^−3^ upon light illumination, indicating around 1.4-fold increment in the supercapacitor charge storage performance. The same is confirmed from the EIS studies, as shown in Fig. 8(c). The value of resistance was decreased upon light irradiation, which further validates the enhanced supercapacitor performance. The solution resistance in the high frequency region was ∼47 Ω in the dark and ∼36 Ω in light. However, these resistance values are way higher than those observed in the case of ReS_2_ on the SS plate, where these values were ∼1.6 Ω for both dark and light conditions, showing the high resistance in the case of the Si/SiO_2_ substrate. Furthermore, it can be clearly seen from the inset of Fig. 8(c) that the charge transfer resistance (CTR) is much higher than that of ReS_2_ on a SS plate. The high resistance observed in the case of ReS_2_ on the Si/SiO_2_ substrate justifies the reason behind its inferior performance as compared to the ReS_2_ on a SS plate, where the current collector is a conducting metal substrate; thus, the charge transfer resistance is decreased in the latter case. This shows that the current collector nature contributes a lot towards the supercapacitor performance of an electrode material. Moreover, the light response of ReS_2_ is perceptible in both cases irrespective of the type of substrate used, and therefore showing the light-fostered supercapacitor performance in both cases. This can be seen in Fig. 9(a) and (b), where the light response is pronounced in all of the GCD curves in both ReS_2_ on Si/SiO_2_, as well as ReS_2_ on a SS plate. Furthermore, the volumetric capacitance values calculated from these GCD curves were plotted as a function of current densities (Fig. 9(c) and (d)), showing the prominent effect of light in the supercapacitor performance in both instances. The detailed electrochemical measurements for ReS_2_ grown on the Si/SiO_2_ substrate in the dark, as well as light, are presented in Fig. S10 and S11, respectively (ESI†).

**Fig. 10:**
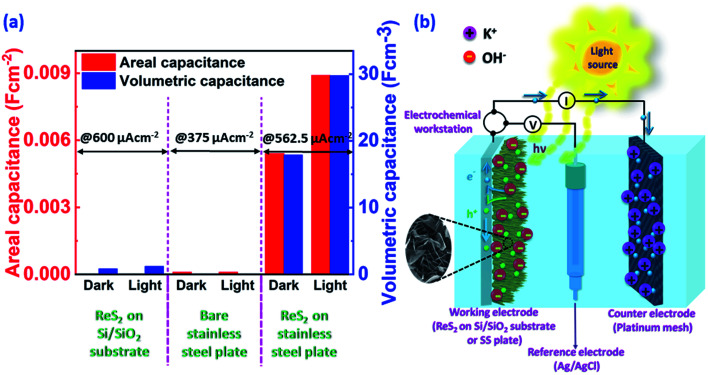
(a) Bar chart shows the light-fostered supercapacitor performance in terms of the areal and volumetric capacitance, and (b) schematic representation of the electron–hole pair generation on light illumination that provides the mechanism for light-induced energy storage in CVD-grown ReS_2_.

The overall comparison of the areal capacitance and volumetric capacitance for ReS_2_ grown on a Si/SiO_2_ substrate, bare SS plate and ReS_2_ grown on a SS plate is shown in the form of a bar chart in Fig. 10(a), which clearly demonstrates the light-fostered charge storage properties of ReS_2_, along with the effect of employing a conducting current collector. This is attributed to the fact that the solution resistance and charge transfer resistance rendered in the case of the SS plate is less than that provided by the non-conducting Si/SiO_2_ substrate. This allows the vertically aligned ReS_2_ to directly and quickly transfer the charge to the conducting current collector in the case of ReS_2_ on a SS plate, unlike ReS_2_ on the Si/SiO_2_ substrate, where the charge carriers have to first transfer from one nanoflower to the other. Where hopping might take place for charge transfer, the connectivity of the ReS_2_ nanoflowers (the growth has to be dense) is required for charge transfer. Furthermore, this charge is then transferred to the electrical circuit aided by silver paste used to make the contact. This reveals the reason behind the better performance of ReS_2_ grown on a SS plate.

The additional effect induced by light is known as photo-charging, which comes into existence due to electron–hole pair generation and their storage within the material in the presence of light. The proposed mechanism of the light effect on the ReS_2_ supercapacitor performance is represented by a schematic in Fig. 10(b). We propose that when the light is incident on the vertically aligned ReS_2_, the electron–hole pair generation transpires. ReS_2_, being a semiconductor,^62–64^ absorbs the light and the photogenerated electron–hole pairs are separated under the applied potential. These electron–hole pairs, being the additional charge carriers, are associated with the energy storage, thereby contributing to the enhanced capacitance. This allows for the accumulation of a large number of electrolyte ions at the interface, thereby increasing the capacitance. The increased current observed in the CV curves can be referred to as the photocurrent. The reason behind the increased charging time on light illumination is that the number of charge carriers is more on the electrode, thereby increasing the number of attracted electrolyte ions. Similarly, the discharging time is increased due to the prolonged separation of the charge-carriers by ReS_2_ or some surface trap states might also play a role; hence, the prolonged recombination time, which leads to an increased discharging time on light illumination.

In order to investigate the effect of the substrate for the photo-charging response, similar experiments have been performed for the bare SS plate in the dark and under light illumination, and are discussed in detail in the ESI in Fig. S12–S14.† Also, to check the temperature effect due to light illumination for this increased supercapacitor performance, we measured the temperature of the cell with the help of a thermometer in light for 30 minutes. No major change has been observed in the temperature, and only 2 degrees of the rise was observed for 30 minutes light irradiation. The results are presented in Table ST2 (ESI†). It should be noted that the actual CV and GCD experiments were performed with the exposure of light for a much shorter duration (around 2 minutes), and hence the temperature effect could be struck out. The presented details confirm that CVD grown vertically oriented ReS_2_ on a conducting substrate is well suited for light-induced enhancement in supercapacitor performance. This opens multiple decisive avenues in the quest of next generation smart energy storage devices with the ability to control the charging–discharging characteristics with the aid of external stimuli, such as light.

## 4. Conclusions

In summary, the vertically oriented single crystalline ReS_2_ with nanoflower-like morphology over the area of 3 cm^2^ and high surface area (as evident from microscopic investigations) was grown on 4 different substrates using chemical vapor deposition (CVD). These ReS_2_ nanoflowers possess a direct band gap of 1.55 eV with multiple transition states. ReS_2_ nanoflowers grown on a non-conducting Si/SiO_2_ substrate and conducting stainless steel plate were used as the electrode material for supercapacitor application. It is important to note that the ReS_2_ nanoflowers grown on a non-conducting Si/SiO_2_ substrate shows inferior supercapacitor performance, in spite of possessing uniform coverage with a high density of vertically aligned ReS_2_ microstructures in comparison to ReS_2_ on a conducting SS plate. This indicates that the high supercapacitor performance of ReS_2_ grown on a SS plate mainly arises from the high conductivity of the SS plate and its interaction with ReS_2_, enabling the easy and quick charge transfer. Under light illumination, a ∼1.7-fold enhancement was observed in the specific capacitance value in terms of the volumetric capacitance due to the extra electron–hole pair generation in the ReS_2_ grown on a SS plate. The demonstrated enhancement is correlated with the photo-charging that paves a new way for the fabrication of smart supercapacitor materials with high specific capacitance value for next generation energy storage devices. Improving the energy storage performance with light radiation warrants further investigation by the right choice of materials and integration of more light-active materials with 2D materials.

## Conflicts of interest

There are no conflicts to declare.

## Supplementary Material

NA-003-D0NA00901F-s001
