# Supplementary material for: A light-fostered supercapacitor performance of multi-layered ReS2 grown on conducting substrates
Source: Nanoscale Adv. 2021 Feb 15;3(7):2089–102. doi: 10.1039/d0na00901f (PMC9419459; doi:10.1039/d0na00901f)
Supplement: NA-003-D0NA00901F-s001 [file NA-003-D0NA00901F-s001.pdf]

Electronic Supplementary Material (ESI) for Nanoscale Advances.
This journal is © The Royal Society of Chemistry 2021

Supplementary Information

Light fostered supercapacitor performance of multi-layered ReS_2 grown on conducting substrates

Nitika Arya, Piyush Avasthi, Viswanath Balakrishnan*

School of Engineering, Indian Institute of Technology, Mandi, Himachal Pradesh, 175005, India

*Corresponding author, E-mail: viswa@iitmandi.ac.in

Table ST1. The Raman active modes and frequencies of all the ReS_2 samples grown on different substrates in the present work.

Table ST2. Proof of concept for the light effect on electrochemical supercapacitor performance of as grown ReS_2 by investigating the temperature effect that might occur on shining the light with time: Table showing the variation of temperature with time on shining light for 30 minutes.

Figure S1. Morphology identification: Low magnification planar FESEM images of ReS_2 grown on (a) FTO coated glass and (b) ITO coated glass. Corresponding high magnification images of ReS_2 showing flower or plates like morphology are shown in the inset of (a) and (b).

Figure S2. Crystallographic identification: (a) Low magnification bright field TEM micrograph showing the electron transparent ultrathin structure of the flowers, (b) HRTEM micrograph of ReS_2 nanostructures along with their SAED pattern in the inset showing the single crystalline nature of as grown ReS_2 on Si/SiO_2 substrate, (c) HRTEM image showing the multi-layer nature of the nanostructures with a 0.6 nm of interlayer distance and (d) X-Ray diffraction pattern of ReS_2 nanoflowers grown on Si/SiO_2 substrate indicating their crystalline nature with preferred orientation of (h00) family reflections.

Figure S3. Elemental composition analysis: XPS spectra of (a) Survey, (b) Re and (c) S showing the elemental composition and chemical states of as grown ReS_2 on Si/SiO_2 substrate.

Figure S4. Electrochemical supercapacitor performance of as grown ReS_2 on SS plate: (a) Cyclic voltammogram at varied scan rates, (b) Galvanostatic charge-discharge cycles at varied current densities and (c) Variation of specific capacitance with respect to current densities.

Figure S5. Cyclic voltammogram of as grown ReS_2 on SS plate with different scan rates from 20 mV/s to 500 mV/s and (b) Capacitive current densities at 0.15 V as a function of scan rate for the estimation of ECSA.

Figure S6. Electrochemical supercapacitor performance of as grown ReS_2 on SS plate in light: (a) Cyclic

voltammogram at varied scan rates, (b) Galvanostatic charge-discharge cycles at varied current densities, (c) Variation of specific capacitance with respect to current densities, (d) Capacitive retention plotted against number of cycles showing ~101% capacitance retention over 1,000 charge-discharge cycles in light; corresponding galvanostatic charge-discharge cycles for 10th and 1000th cycle are shown in the inset, (e) Cyclic voltammetry curves before and after cyclic stability and (f) Nyquist plot before and after cyclic stability.

Figure S7. (a) Galvanostatic charge-discharge cycles, (b) Five GCD cycles showing the reversible nature of light effect on charging-discharging cycle in dark and light.

Figure S8. Electrochemical supercapacitor performance of as grown ReS_2 on SS plate in dark and light in terms of coulombic efficiency.

Figure S9. Electrochemical supercapacitor performance of as grown ReS_2 on SS plate in dark and light conditions to avoid electrode polarization effect by reducing the potential range of 0 to -0.5 V: (a) Cyclic voltammogram at varied scan rates in dark, (b) Galvanostatic charge-discharge cycles at varied current densities in dark, (c) Coulombic efficiency plotted as a function of cycle number in dark, (d) Cyclic voltammogram at varied scan rates in light, (e) Galvanostatic charge-discharge cycles at varied current densities in light, (f) Coulombic efficiency plotted as a function of cycle number in light.

Figure S10. Electrochemical supercapacitor performance of as grown ReS_2 on Si/SiO_2 substrate in dark condition: (a) Cyclic voltammogram at varied scan rates, (b) Galvanostatic charge-discharge cycles at varied current densities, (c) Nyquist plot showing high resistance, (d) Variation of specific capacitance as a function of current densities, (d) GCD curves of 10th and 1000th cycle showing the high capacitance retention (e) of 93.75%, (e & f) CV curves and Nyquist plots conducted before and after cyclic stability supporting the good cyclic stability results.

Figure S11. Electrochemical supercapacitor performance of as grown ReS_2 on Si/SiO_2 substrate upon illumination of light: (a) Cyclic voltammogram at varied scan rates, (b) Galvanostatic charge-discharge cycles at varied current densities, (c) Nyquist plot showing high resistance, (d) Variation of specific capacitance as a function of current densities, (d) GCD curves of 10th and 1000th cycle showing the high capacitance retention (e) of 93.75%, (e & f) CV curves and Nyquist plots conducted before and after cyclic stability supporting the good cyclic stability results.

Figure S12. Electrochemical supercapacitor performance of bare SS plate in dark: (a) Cyclic voltammogram taken at varied scan rates, (b) Galvanostatic charge-discharge cycles at varied current densities and (c) Electrochemical impedance spectra (Nyquist plot).

Figure S13. Electrochemical supercapacitor performance of bare SS plate in light: (a) Cyclic voltammogram taken at varied scan rates, (b) Galvanostatic charge-discharge cycles at varied current densities and (c) Electrochemical impedance spectra (Nyquist plot).

Figure S14. Comparative electrochemical supercapacitor performance of bare SS plate in dark (black) and light (red): (a) Cyclic voltammogram taken at 100 mV/s scan rate, (b) Galvanostatic charge-discharge cycles at varied current densities and (c) Electrochemical impedance spectra showing almost similar behaviour in dark as well as in light.

Raman Spectroscopy:

ReS_2 grown on all the substrates; conducting as well as non-conducting substrates has been characterized by Raman spectroscopy. The excitation wavelength of laser used for the spectroscopy is 532 nm. We have tabulated the frequencies and Raman active modes of the obtained peaks for all four samples in table ST1 and compared them with the reported experimental values by Feng et al. [1] for bulk ReS_2 at excitation wavelength laser of 633 nm (a) and by He et al. [2] at 532 nm excitation wavelength laser (b). The Raman active modes and frequencies obtained in the present work are congruent with earlier reported values.

Table ST1. The Raman active modes and frequencies of all the ReS_2 samples grown on different substrates in the present work.

Symmetry [a]	Attributes to [a]	Reference [a]	Reference [b]	Our samples on different substrates			
				Si/SiO_2	FTO	ITO	SSP
		Raman shift (cm^{-1})	Raman shift (cm^{-1})	Raman shift (cm^{-1})	Raman shift (cm^{-1})	Raman shift (cm^{-1})	Raman shift (cm^{-1})
A_g-like	Out-of-plane vibrations of Re atoms	140.3	135.2	136.2	136.4	136.4	135.9
A_g-like		145.9	141.8	142.0	141.1	141.6	141.1
E_g-like	In-plane vibrations of Re atoms	153.1	150.4	150.8	150.4	149.9	149.4
E_g-like		163.6	160.9	160.6	160.3	160.3	159.2
E_g-like		217.2	211.6	212.3	211.8	210.8	210.3
E_g-like		237.1	234.7	234.9	233.8	233.3	232.8
C_p	In-plane and out-of-plane vibrations of Re & S atoms	278.3	275.8	275.6	274.1	274.1	274.1
C_p		284.2	282.9	282.1	281.2	280.7	279.2
E_g-like	In-plane vibrations of S atoms	307.8	305.8	308.2	304.6	305.1	304.1
E_g-like		311.0		311.6	312.7	312.2	312.2
C_p	In-plane and out-of-plane vibrations of S atoms	320.6	319.2	317.8	316.7	317.7	316.7
C_p		324.9	324.2	323.4	322.3	321.8	320.7
C_p		348.8	346.8	347.0	346.4	344.9	344.4
C_p		368.9	368.7	367.4	367.0	367.0	365.0
C_p		377.9	375.1	376.1	375.5	375.0	374.5
C_p		407.3	407.1	406.9	405.5	405.0	404.5
A_g-like	Out-of-plane vibrations of S atoms	418.7	417.4	417.6	416.4	415.4	415.4
A_g-like		438.0	435.5	435.9	434.8	434.3	433.8

Temperature effect upon light illumination:

To probe the possible effect of temperature rise during light illumination on the electrochemical supercapacitor performance, we have studied the variation of temperature with time on shining the light for 30 minutes as shown in table ST2. We observed a small change of two degrees only and hence, eliminating the temperature effect on electrochemical measurements upon light illumination. Note that the actual electrochemical measurements are done with illumination of light within 2 minutes.

Table ST2. Proof of concept for the light effect on electrochemical supercapacitor performance of as grown ReS_2 by investigating the temperature effect that might occur on shining the light with time: Table showing the variation of temperature with time on shining light for 30 minutes.

Time (minutes)	Temperature (°C)
**0**	30
**5**	30
**10**	30
**15**	31
**20**	31
**25**	32
**30**	32

*** All measurements are done in 1 M KOH with a dipped thermometer. Light source used here is 150 W Xenon lamp (Holmarc) ranging from 200 to 2500 nm wavelength.**

FESEM micrographs of ReS_2 grown on FTO coated glass and ITO coated glass:

The low magnification FESEM micrographs for ReS_2 grown on FTO coated glass and ITO coated glass are presented in figure S1 (a & b) showing the variation in aerial coverage of ReS_2 growth. The corresponding high magnification FESEM micrographs are shown in the inset of figure S1 (a & b). This indicates the substrate dependence on growth of ReS_2 and its controlled growth on four different substrates including both conducting and non-conducting substrates.

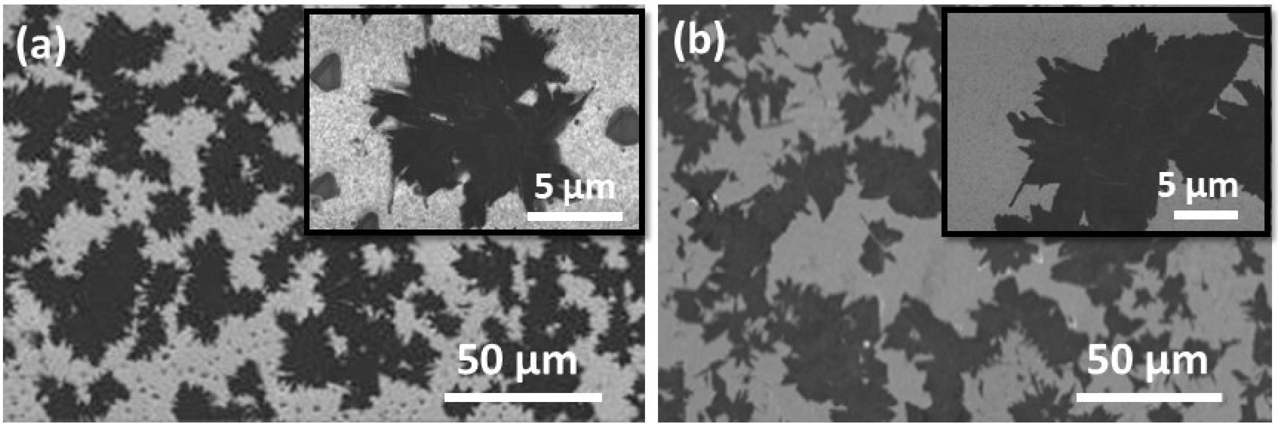

Figure S1. Morphology identification: Low magnification planar FESEM images of ReS_2 grown on (a) FTO coated glass and (b) ITO coated glass. Corresponding high magnification images of ReS_2 showing flower or plates like morphology are shown in the inset of (a) and (b).

TEM micrographs and XRD pattern of ReS_2 grown on Si/SiO_2 substrate:

Bright field TEM micrograph in figure S2 (a) shows the electron transparent ultrathin structure of ReS_2 nanoflowers at low magnification. Figure S2 (b) shows the HRTEM image along with the corresponding selected area electron diffraction (SAED) with spot pattern shown in the inset. HRTEM micrograph presented in figure S2 (c), provides an impression regarding the number of layers of as grown nanoflowers of ReS_2. Further, to verify the single crystalline nature of ReS_2, X-Ray diffraction analysis was carried out as shown in figure S2 (d).

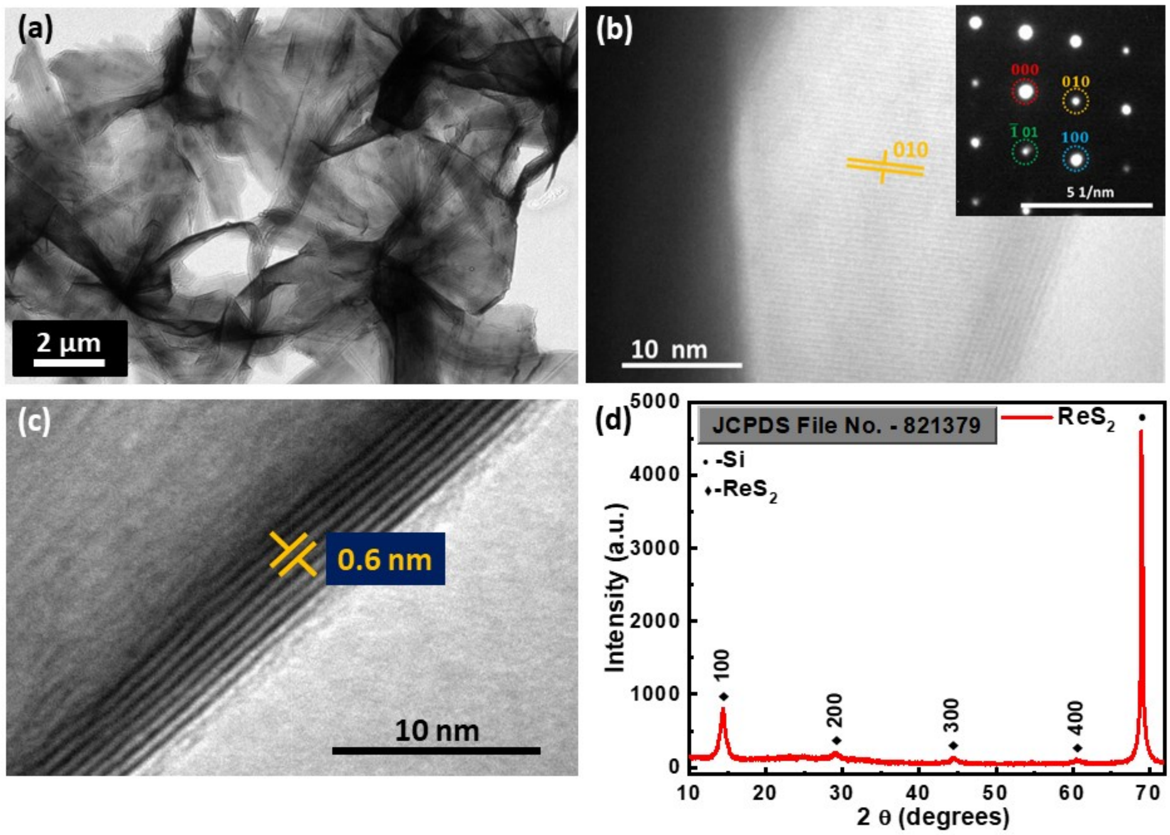

Figure S2. Crystallographic identification: (a) Low magnification bright field TEM micrograph showing the electron transparent ultrathin structure of the flowers, (b) HRTEM micrograph of ReS_2 nanostructures along with their SAED pattern in the inset showing the single crystalline nature of as grown ReS_2 on Si/SiO_2 substrate, (c) HRTEM image showing the multi-layer nature of the nanostructures with a 0.6 nm of interlayer distance and (d) X-Ray diffraction pattern of ReS_2 nanoflowers grown on Si/SiO_2 substrate indicating their crystalline nature with preferred orientation of (h00) family reflections.

XPS analysis of ReS_2 grown on Si/SiO_2 substrate:

Figure S3 shows the XPS results which provides a crux of elemental composition and their chemical states in ReS_2 nanoflowers grown on Si/SiO_2 substrate. Figure S3 (a) displays the survey spectrum of as grown ReS_2 nanoflowers, which shows the presence of four elements, Re and S from ReS_2 and Si and O from Si/SiO_2. The two peaks appearing in XPS spectrum for Re in figure S3 (b) attributes Re^{4+} oxidation state. The XPS spectrum of S is shown in figure S3 (c) and possesses two peaks ascribing to sulphide (S^{-2}) ions.

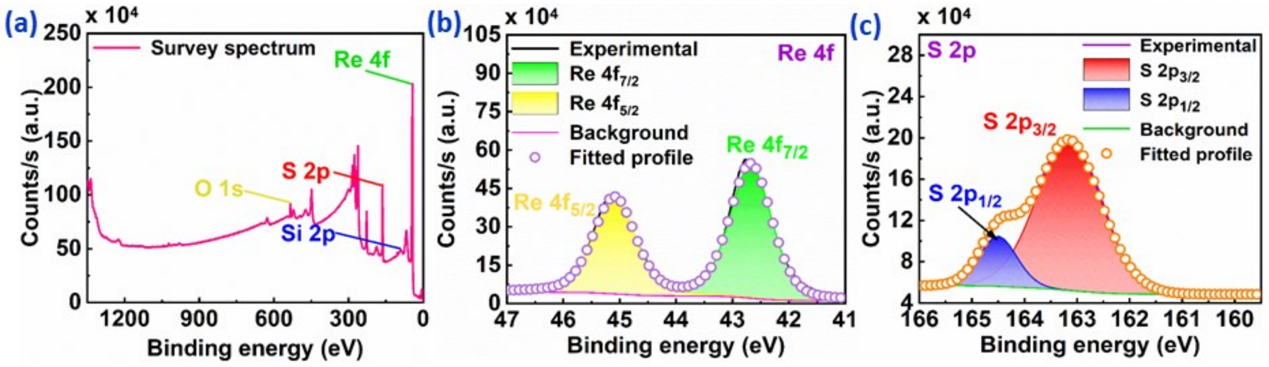

Figure S3. Elemental composition analysis: XPS spectra of (a) Survey, (b) Re and (c) S showing the elemental composition and chemical states of as grown ReS_2 on Si/SiO_2 substrate.

Electrochemical measurements for ReS_2 grown on SSP substrate in dark:

The detailed electrochemical measurements for ReS_2 grown on SS plate in dark conditions are shown in figure S4, where figure S4 (a) shows the cyclic voltammograms at varied scan rates of 20, 50, 100, 200 and 500 mV/s. The predominant EDLC behaviour can clearly be observed along with subsidiary characteristics of pseudocapacitive behaviour mainly between -0.2 to -0.4 V potential region. Figure S4 (b) shows the galvanostatic charging-discharging curves at varied current densities ranging from 562.5 μAcm^{-2} to 937.5 μAcm^{-2}. These GCD curves has been used to calculate the specific capacitance values by using equation 4 (expressed in main paper). The specific capacitance values thus obtained are plotted with respect to the corresponding scan rate values as represented in figure S4 (c).

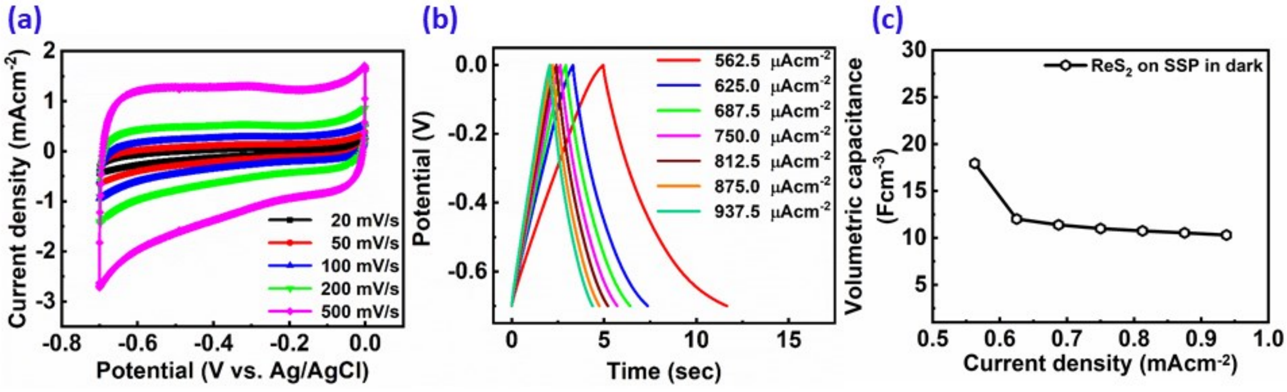

Figure S4. Electrochemical supercapacitor performance of as grown ReS_2 on SS plate: (a) Cyclic voltammogram at varied scan rates, (b) Galvanostatic charge-discharge cycles at varied current densities and (c) Variation of specific capacitance with respect to current densities.

Electrochemical active surface area of ReS_2 grown on stainless steel plate:

The cyclic voltammetry experiments were performed in a potential window of -0.1 V to -0.2 V at different scan rates starting from 20 mV/s to 500 mV/s for calculating the Electrochemical active Surface Area (ECSA) as shown in figure S5 (a). The difference in current density (Δj) in middle of the potential range @ 0.15 V divided by 2 is plotted as a function of scan rate. The data points thus obtained were fitted linearly to obtain the value of the slope as shown in figure S5 (b). This value of slope thus obtained is C_{dl}, the double layer capacitance of our working electrode. This double layer capacitance (C_{dl}) value is further used to calculate the roughness factor (R_f), by using the following equation[3–5]:

$$R_f = \frac{C_{dl}}{C_s} \qquad \textbf{.....(1)}$$

where Cs is the average double layer capacitance of a smooth metal surface which is assumed to be 20 μFcm^{-2}. Here, the value of C_{dl} comes out to be 1,410 μFcm^{-2}. Therefore, the estimated value of roughness factor is found to be 70.5. This roughness factor is then used to estimate the ECSA of our working electrode by using the following equation

$$ECSA = R_f\, S \qquad \textbf{.....(2)}$$

where S denotes the actual surface area of the smooth metal electrode, which here is the geometric area of our working electrode that was dipped into the electrolyte i.e. 0.8 cm^2. Therefore, ECSA thus calculated is found to be 56.4 cm^2. This manifests that a high surface area of 56.4 cm^2 is provided for charge storage by the beautiful vertically aligned microstructure of ReS_2 just within a small geometrical area of 0.8 cm^2.

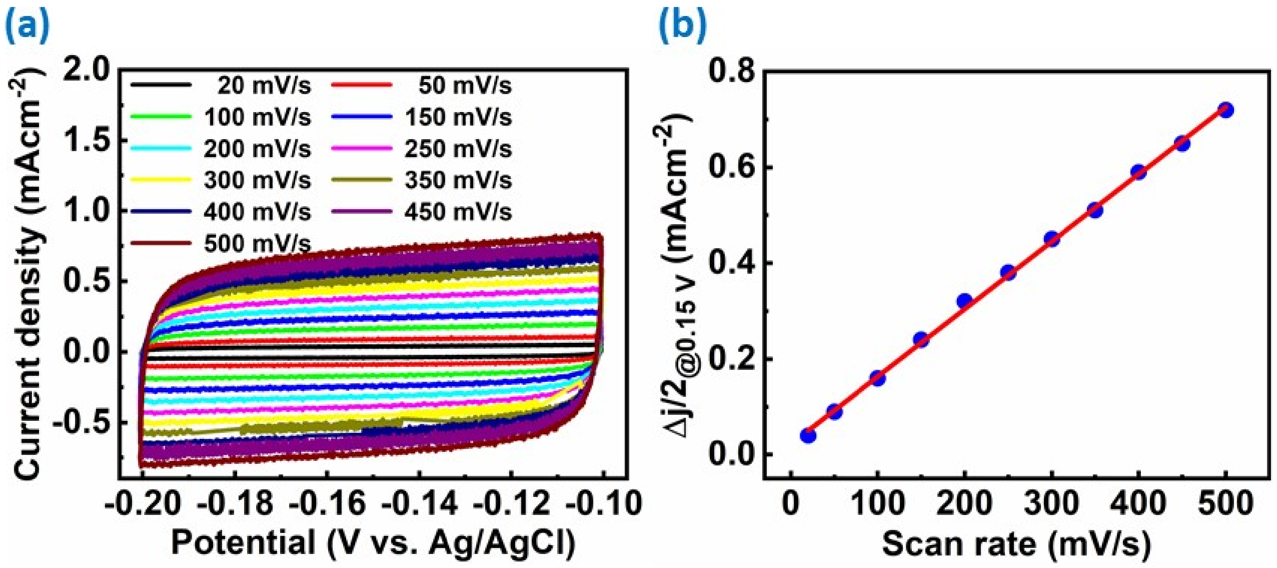

Figure S5. Cyclic voltammogram of as grown ReS_2 on SS plate with different scan rates from 20 mV/s to 500 mV/s and (b) Capacitive current densities at 0.15 V as a function of scan rate for the estimation of ECSA.

Electrochemical measurements for ReS_2 grown on SS plate in light:

The detailed electrochemical properties of ReS_2 grown on SS plate upon light illumination are shown in figure S6, where figure S6 (a) shows the cyclic voltammograms at varied scan rates of 20, 50, 100, 200 and 500 mV/s showing the primary contribution from EDL and a small pseudocapacitive behaviour of ReS_2. Figure S6 (b) shows the galvanostatic charge-discharge curves at varied current densities ranging from 562.5 μAcm^{-2} to 937.5 μAcm^{-2}. The estimated volumetric capacitance is plotted as a function of varying current density as presented in figure S6 (c). We performed cyclic stability test upto 1000 charge-discharge cycles in light condition and the electrode showed excellent cyclic stability by retaining its initial capacitance as shown by figure S6 (d). This stability of electrode is evident from the comparative charge-discharge curves of 10th and 1000th cycle shown in the inset of figure S6 (d), the CV curves and Nyquist plots taken before and after 1000 GCD cycles as represented in figures S6 (e & f).

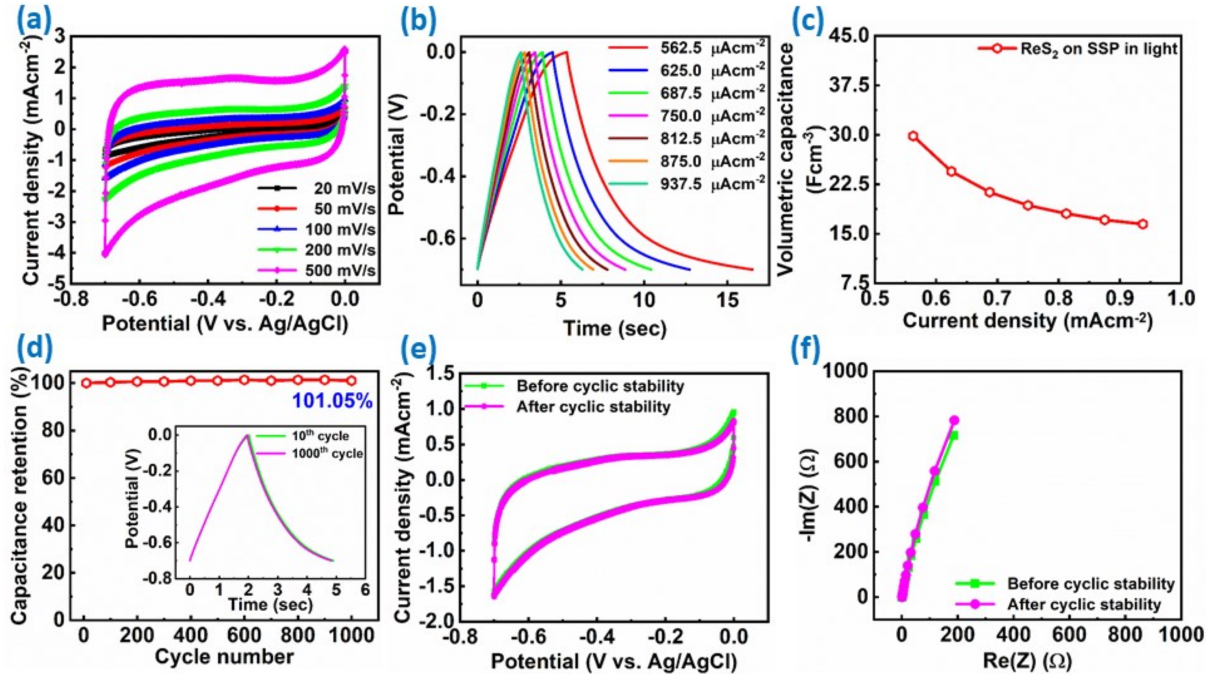

Figure S6. Electrochemical supercapacitor performance of as grown ReS_2 on SS plate in light: (a) Cyclic voltammogram at varied scan rates, (b) Galvanostatic charge-discharge cycles at varied current densities, (c) Variation of specific capacitance with respect to current densities, (d) Capacitive retention plotted against number of cycles showing ~101% capacitance retention over 1,000 charge-discharge cycles in light; corresponding galvanostatic charge-discharge cycles for 10th and 1000th cycle are shown in the inset, (e) Cyclic voltammetry curves before and after cyclic stability and (f) Nyquist plot before and after cyclic stability.

Galvanostatic charge discharge curves for ReS_2 grown on SS plate in light at lower current density:

The galvanostatic charge discharge curves for ReS_2 grown on SS plate in light at lower current density of 375 µAcm^{-2} are shown in figure S7 (a). The specific capacitance values calculated from GCD curves found to be 21,066 mFcm^{-3} in dark and 70,366 mFcm^{-3} upon illumination with light. This clearly reveals the effect of light on supercapacitor charge storage performance by exhibiting an enhancement of ~3 folds in specific capacitance values upon light illumination. The corresponding five charging-discharging cycle in dark (black) and light (red) are shown in figure S7 (b). But there is a clear signature of pronounced electrode polarization beyond -0.5 V potential, which can have a negative impact on the charge storage performance of the electrode under consideration in terms of efficiency.

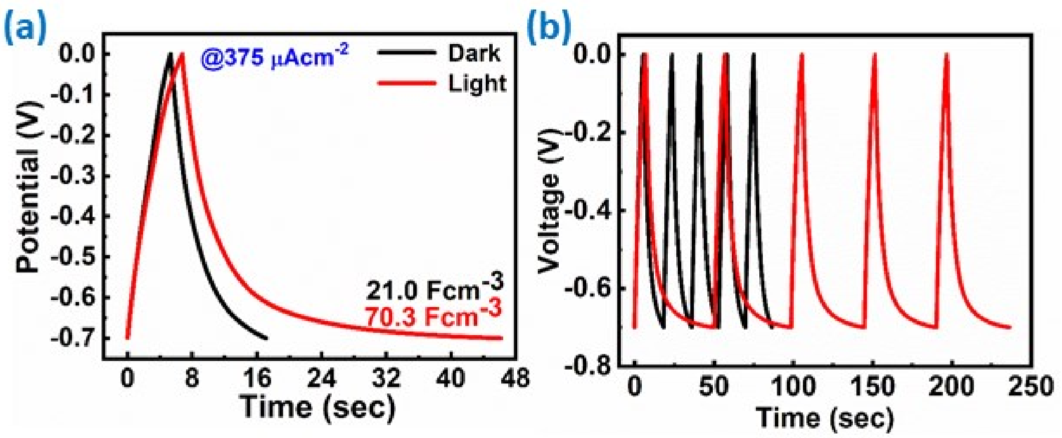

Figure S7. (a) Galvanostatic charge-discharge cycles, (b) Five GCD cycles showing the reversible nature of light effect on charging-discharging cycle in dark and light.

Coulombic efficiency of ReS_2 grown on SS plate in dark and light:

The coulombic efficiency was calculated for as grown ReS_2 on SS plate in dark and light. The GCD curves at 562.5 µAcm^{-2} current density were used for estimating the efficiency. The GCD curves shows the non-linear characteristics and following expression was employed for the calculation of coulombic efficiency (η):

$$\eta = \int \frac{E \times t_{discharging}}{E \times t_{charging}} X\,100 \quad \text{.....(3)}$$

where $\int E \times t_{dicharging}$ refers to the integral of area under the discharging curve and $\int E \times t_{charging}$ refers to the integral of area under the charging curve. The coulombic efficiency thus calculated came out to be around 77.8% in dark and around 71.3% in light. This is presented in figure S8. The reason behind such coulombic efficiency is the non-ideal behaviour GCD curves arising due to some faradaic reaction contribution as it is not showing the pure EDL behaviour.

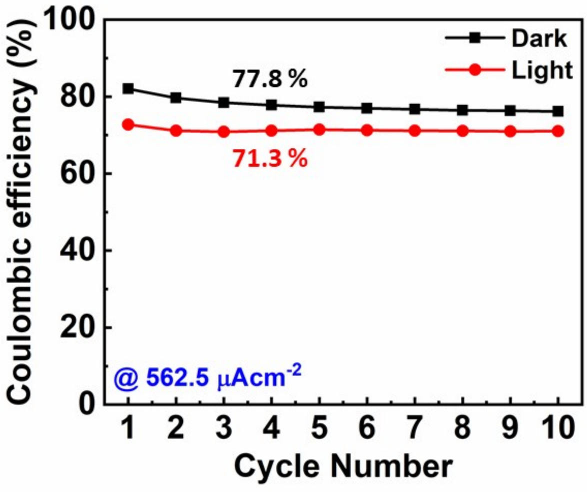

Figure S8. Electrochemical supercapacitor performance of as grown ReS_2 on SS plate in dark and light in terms of coulombic efficiency.

As evident from figure 6 (d), a considerable electrode polarization occurs at a potential higher than -0.5 V. This can also result in lower coulombic efficiency. In order to avoid such undesirable and detrimental effect of electrode polarization, we limit the potential range of galvanostatic charge discharge curves from 0 to -0.5 V. The detailed electrochemical measurements are shown in figure S9, which elucidates the disappearance of electrode polarization after reducing the potential window up to -0.5 V. Moreover, the increase in coulombic efficiency after decreasing the potential window was also observed. The coulombic efficiency thus calculated comes out to be ~90 % in dark and 88 % in light.

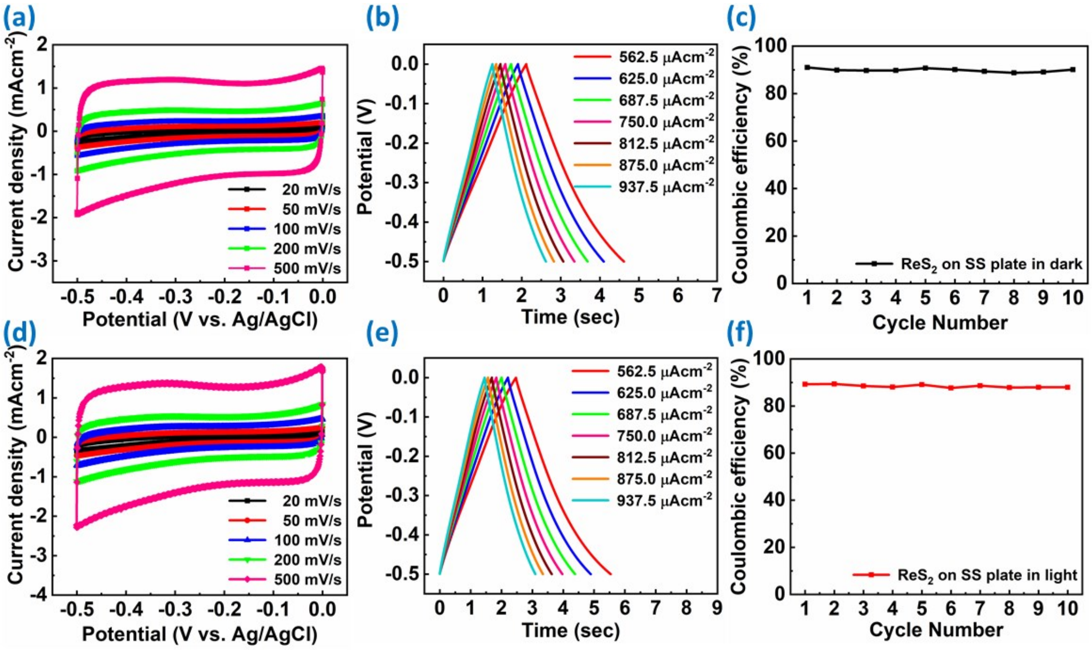

Figure S9. Electrochemical supercapacitor performance of as grown ReS_2 on SS plate in dark and light conditions to avoid electrode polarization effect by reducing the potential range of 0 to -0.5 V: (a) Cyclic voltammogram at varied scan rates in dark, (b) Galvanostatic charge-discharge cycles at varied current densities in dark, (c) Coulombic efficiency plotted as a function of cycle number in dark, (d) Cyclic voltammogram at varied scan rates in light, (e) Galvanostatic charge-discharge cycles at varied current densities in light, (f) Coulombic efficiency plotted as a function of cycle number in light.

The specific capacitance values calculated at a current density of 562.5 μAcm^{-2} for dark and light comes out to be 9.4 Fcm^{-3} and 11.5 Fcm^{-3}.

Electrochemical measurements for ReS_2 grown on Si/SiO_2 substrate in dark and light:

The detailed electrochemical properties of ReS_2 grown on Si/SiO_2 substrate in dark and light conditions are shown in figure S10 and figure S11, where figure S10 (a) and figure S11 (a) shows the cyclic voltammograms at varied scan rates of 20, 50, 100, 200 and 500 mV/s in dark and light respectively. The cyclic voltammograms in light and dark manifest the contribution from both EDL and pseudocapacitive behaviour of ReS_2 grown on Si/SiO_2 substrates. Figure S10 (b) and figure S11 (b) shows the galvanostatic charge-discharge curves at varied current densities ranging from 600 μAcm^{-2} to 900 μAcm^{-2} in dark and light, respectively. The estimated volumetric capacitance is plotted as a function of varying current density for both dark and light in figure S10 (d) and figure S11 (d). The electrochemical impedance spectroscopy has been performed and the corresponding Nyquist plot are represented in figure S10 (c) and figure S11 (c) for dark and light. We performed cyclic stability test upto 1000 charge-discharge cycles for ReS_2 grown on Si/SiO_2 substrate under dark condition as well as upon light illumination. The electrode showed excellent cyclic stability of > 90% in both dark and light conditions as evident from the comparative charge-discharge curves of 10th and 1000th cycle, capacitance retention plot as a function of GCD cycle number, CV curves and Nyquist plots taken before and after 1000 GCD cycles for both dark and light conditions as represented in figures S10 (e-h) and figures S11 (e-h).

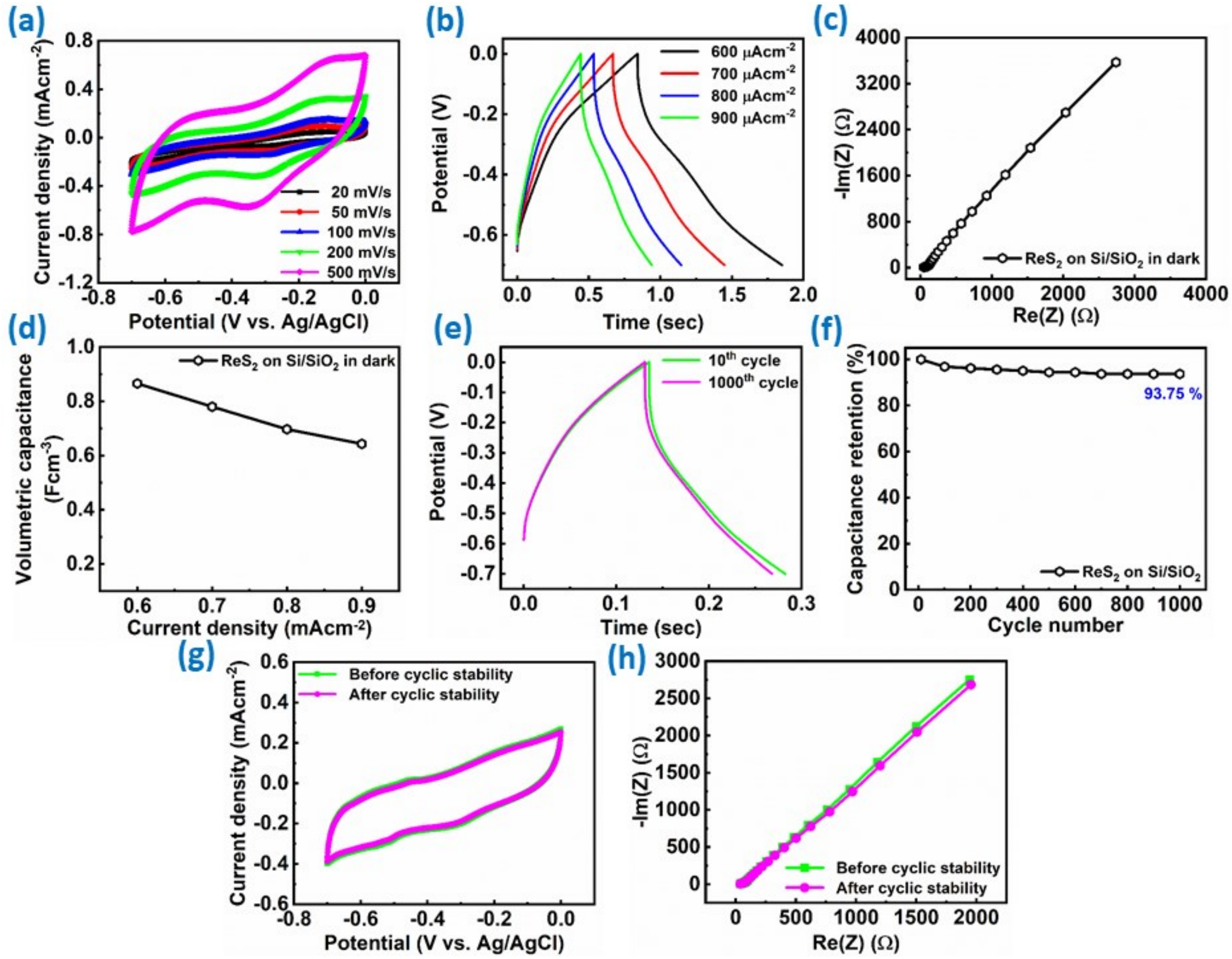

Figure S10. Electrochemical supercapacitor performance of as grown ReS_2 on Si/SiO_2 substrate in dark condition: (a) Cyclic voltammogram at varied scan rates, (b) Galvanostatic charge-discharge cycles at varied current densities, (c) Nyquist plot showing high resistance, (d) Variation of specific capacitance as a function of current densities, (d) GCD curves of 10th and 1000th cycle showing the high capacitance retention (e) of 93.75%, (e & f) CV curves and Nyquist plots conducted before and after cyclic stability supporting the good cyclic stability results.

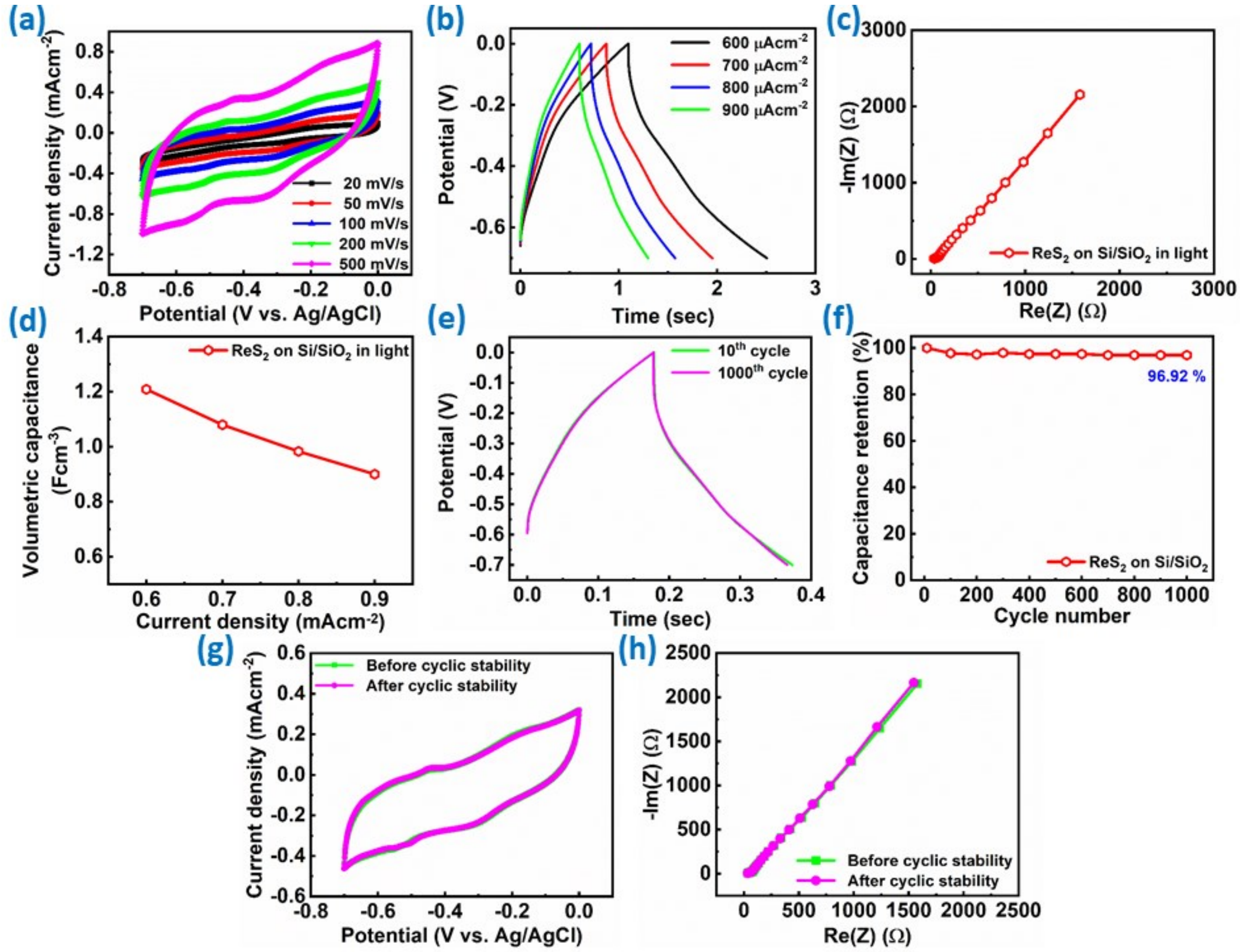

Figure S11. Electrochemical supercapacitor performance of as grown ReS_2 on Si/SiO_2 substrate upon illumination of light: (a) Cyclic voltammogram at varied scan rates, (b) Galvanostatic charge-discharge cycles at varied current densities, (c) Nyquist plot showing high resistance, (d) Variation of specific capacitance as a function of current densities, (d) GCD curves of 10th and 1000th cycle showing the high capacitance retention (e) of 93.75%, (e & f) CV curves and Nyquist plots conducted before and after cyclic stability supporting the good cyclic stability results.

Electrochemical supercapacitor performance of bare SS plate in dark:

The detailed electrochemical measurements for bare SS plate in dark conditions are shown in figure S12, where figure S12 (a) shows the cyclic voltammograms at varied scan rates of 20, 50, 100, 200 and 500 mV/s. Figure S12 (b) shows the galvanostatic charging-discharging curves at varied current densities ranging from 126.20 μAcm^{-2} to 584.79 μAcm^{-2}. It is manifested from figure S12 (b) that the charging and discharging are taking place in fraction of seconds. The electrochemical impedance spectroscopy has been performed and the corresponding Nyquist plot is represented in figure S12 (c).

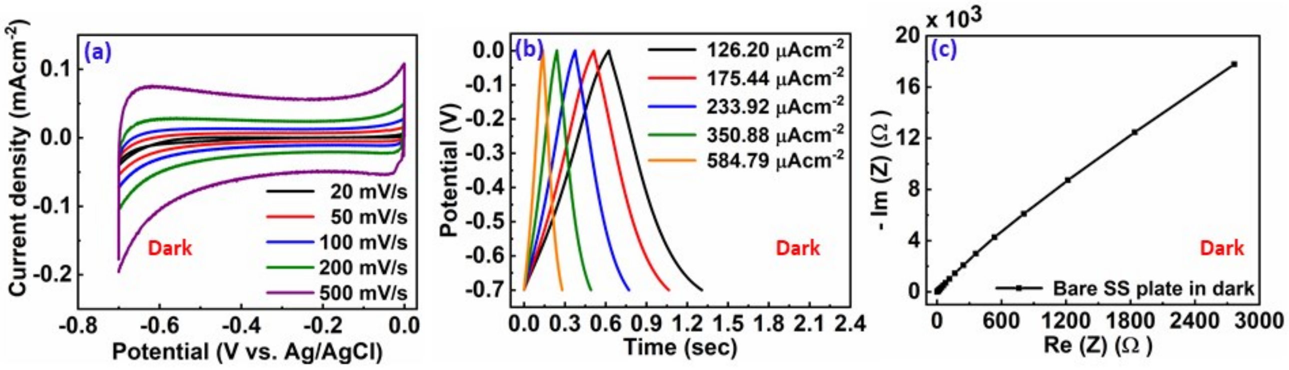

Figure S12. Electrochemical supercapacitor performance of bare SS plate in dark: (a) Cyclic voltammogram taken at varied scan rates, (b) Galvanostatic charge-discharge cycles at varied current densities and (c) Electrochemical impedance spectra (Nyquist plot).

Electrochemical supercapacitor performance of bare SS plate in light:

The detailed electrochemical measurements for bare SS plate in light conditions are shown in figure S13, where figure S13 (a) shows the cyclic voltammograms at varied scan rates of 20, 50, 100, 200 and 500 mV/s. Figure S13 (b) shows the galvanostatic charging-discharging curves at varied current densities varying from 126.20 μAcm^{-2} to 584.79 μAcm^{-2}. Here also, the charging and discharging are taking place very rapidly just in fraction of seconds. The electrochemical impedance spectroscopy has been performed and the corresponding Nyquist plot is represented in figure S13 (c).

Figure S13. Electrochemical supercapacitor performance of bare SS plate in light: (a) Cyclic voltammogram taken at varied scan rates, (b) Galvanostatic charge-discharge cycles at varied current densities and (c) Electrochemical impedance spectra (Nyquist plot).

Comparative electrochemical supercapacitor performance of bare SS plate under dark and light conditions:

The electrochemical supercapacitor performance of bare SS plate has been compared in dark and light conditions to eliminate the substrate effect in light-induced supercapacitor performance. Figure S14 (a) shows the cyclic voltammograms at 100 mV/s scan rate. Figure S14 (b) shows the galvanostatic charge-discharge curves at 350.88 μAcm^{-2} current density. The electrochemical impedance spectroscopy has been performed and the corresponding comparative Nyquist plots are represented in figure S14 (c). There is no prominent difference observed in dark and light conditions in any of the electrochemical measurements, thereby, manifesting that the bare SS plate is not absorbing the light and hence, no light effect can be observed in it. Therefore, eliminating the effect of substrate in light induced supercapacitor performance of ReS_2 grown on SS plate.

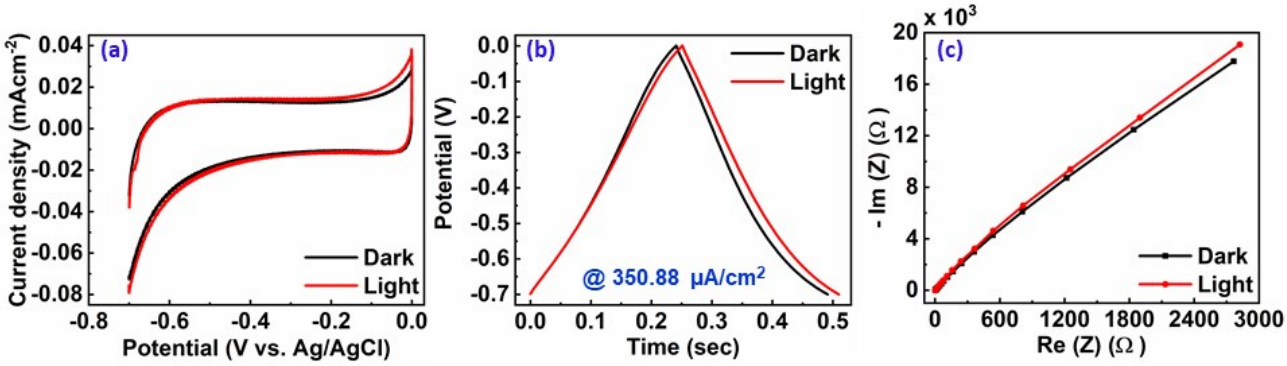

Figure S14. Comparative electrochemical supercapacitor performance of bare SS plate in dark (black) and light (red): (a) Cyclic voltammogram taken at 100 mV/s scan rate, (b) Galvanostatic charge-discharge cycles at varied current densities and (c) Electrochemical impedance spectra showing almost similar behaviour in dark as well as in light.

References:

1 Y. Feng, W. Zhou, Y. Wang, J. Zhou, E. Liu, Y. Fu, Z. Ni, X. Wu, H. Yuan, F. Miao, B. Wang, X. Wan and D. Xing, *Phys. Rev. B*, 2015, **92**, 1–23.

2 R. He, J. A. Yan, Z. Yin, Z. Ye, G. Ye, J. Cheng, J. Li and C. H. Lui, *Nano Lett.*, 2016, **16**, 1404–1409.

3 S. Trasatti and O. A. Petrii, *Pure Appl. Chem.*, 1991, **63**, 711–734.

4 J. Sen Li, Y. Wang, C. H. Liu, S. L. Li, Y. G. Wang, L. Z. Dong, Z. H. Dai, Y. F. Li and Y. Q. Lan, *Nat. Commun.*, 2016, **7**, 1–8.

5 S. Gao, Y. Lin, X. Jiao, Y. Sun, Q. Luo, W. Zhang, D. Li, J. Yang and Y. Xie, *Nature*, 2016, **529**, 68–71.
